# Discovery, Replicability, and Generalizability of a Left Anterior Hippocampus' Morphological Network Linked to Self‐Regulation

**DOI:** 10.1002/hbm.70099

**Published:** 2024-12-20

**Authors:** Somayeh Maleki Balajoo, Anna Plachti, Eliana Nicolaisen‐Sobesky, Debo Dong, Felix Hoffstaedter, Sven G. Meuth, Nico Melzer, Simon B. Eickhoff, Sarah Genon

**Affiliations:** ^1^ Institute of Systems Neuroscience Heinrich Heine University Düsseldorf Düsseldorf Germany; ^2^ Institute of Neuroscience and Medicine (INM‐7: Brain and Behaviour) Research Centre Jülich Jülich Germany; ^3^ Key Laboratory of Cognition and Personality, Ministry of Education, Faculty of Psychology Southwest University Chongqing China; ^4^ Department of Neurology, Medical Faculty and University Hospital Düsseldorf Heinrich Heine University Düsseldorf Düsseldorf Germany

**Keywords:** brain–behavior relationships, CCA, generalizability, hippocampus' organization, machine learning, replicability

## Abstract

The human hippocampus is a key region in cognitive and emotional processing, but also a vulnerable and plastic region. Accordingly, there is a great interest in understanding how variability in the hippocampus' structure relates to variability in behavior in healthy and clinical populations. In this study, we aimed to link interindividual variability in subregional hippocampal networks (i.e., the brain grey matter networks of hippocampal subregions) to variability in behavioral phenotype. To do so, we used a multiblock multivariate approach mapping the association between grey matter volume in hippocampal subregions, grey matter volume in the whole brain regions, and behavioral variables in healthy adults. To ensure the robustness and generalizability of the findings, we implemented a cross‐cohort discovery and validation framework. This framework utilized two independent cohorts: the Human Connectome Project Young Adult (HCP‐YA) cohort and the Human Connectome Project Aging (HCP‐A) cohort, enabling us to assess the replicability and generalizability of hippocampal–brain–behavior phenotypes across different age groups in the population. Our results highlighted a left anterior hippocampal morphological network including the left amygdala and the posterior midline structures whose expression relates to higher self‐regulation, life satisfaction, and better performance at standard neuropsychological tests. The cross‐cohort generalizability of the hippocampus–brain–behavior mapping demonstrates its relevance beyond a specific population sample. Our previous work in developmental populations showed that the hippocampus' head co‐maturates with most of the brain during childhood. The current data‐driven study further suggests that grey matter volume in the left hippocampal head network would be particularly relevant for self‐regulation abilities in adults that influence a range of life outcomes. Future studies should thus investigate the factors influencing the development of this morphological network across childhood, as well as its relationship to neurocognitive phenotypes in various brain diseases.

## Graphical abstract

We delineate the replicable and generalizable relationship between grey matter volume in the different hippocampal networks and behavioral phenotypes in a healthy population by implementing a cross‐cohort discovery and validation framework. The different hippocampal networks correspond to the brain regions associated with three different hippocampal subregions: the head, the medial, and the lateral posterior region. To disentangle robust patterns, we used a novel multiblock multivariate approach within a machine learning framework within two adult cohorts (HCP young adult and HCP aging). We hence identified a stable and significant latent pattern highlighting specifically a left anterior hippocampal morphological network encompassing the left amygdala and posterior midline cortical structures and that robustly relates to self‐regulation abilities, life satisfaction, and cognitive control performance.

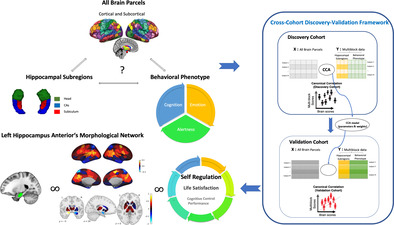

## 1 Introduction

The hippocampus is a key region in cognitive and emotional processing, but also a vulnerable and plastic region. Accordingly, there is a great interest in understanding how variability in the hippocampus' structure relates to variability in behavior in healthy and diseased human populations. Although many previous studies have reported mapping between the hippocampus' morphometry and specific behavioral performance or traits, such one‐to‐one mapping was shown to be affected by replicability issues (Genon, Eickhoff, and Kharabian, 2022; Kharabian Masouleh et al. 2019, 2022; Kharabian Masouleh et al. 2022). For example, Clark et al. recently showed that relationships between hippocampal volume and cognitive performance could hardly be evidenced in a sample of healthy adults of moderate size although many studies in the past have reported specific associations (Clark et al. 2020). To understand this issue, several limitations of the usually taken approaches have to be considered in light of current conceptual and methodological considerations about brain organization and brain–behavior relationships.

First, it is now widely acknowledged that the hippocampus is a heterogeneous region integrated into several functional systems, subserving different behavioral phenotypes (Genon et al. 2021; Genon et al. 2018; Plachti et al. 2019). This engagement typically varies across the anterior–posterior axis (Plachti et al. 2019; Kharabian Masouleh et al. 2020). Generally, anterior hippocampal regions (“hippocampus' head”) appear functionally coupled with a self‐centric system, while posterior regions are more engaged in an action‐oriented system (Genon et al. 2021; Plachti et al. 2019; Maleki Balajoo et al. 2023). These patterns can be observed in functional connectivity data from fMRI (see (Plachti et al. 2019)), but also in co‐morphology between brain regions as studied with structural covariance in grey matter volume (Plachti et al. 2019, 2020; Plachti et al. 2020; Kharabian Masouleh et al. 2020). In that regard, our recent work has suggested that the anterior hippocampus co‐maturates with most of the brain in childhood, while the posterior hippocampus covaries with cortical development at later developmental stages, starting mainly in adolescence (Plachti et al. 2023). Accordingly, the expression of a given behavioral phenotype in an individual will depend on the structural/morphological pattern of different subregional hippocampal–brain networks.

Second, brain–behavior relationships are typically multivariate and this would be particularly the case for highly connected regions such as hippocampal subregions. Since, as aforementioned, grey matter volume in each hippocampal subregion covaries with a range of other brain regions across individuals, considering the whole morphological (i.e., grey matter covariance) network in association with behavior may be conceptually more enlightening and methodologically more powerful than searching for specific associations between grey matter volume within the hippocampus and behavioral variables. Furthermore, it appears from activations studies that hippocampal subregions are engaged in a range of behavioral paradigms, thus a range of behavioral variables could be considered when aiming to relate hippocampal grey matter networks to behavioral measurements. Additionally, from a psychometric data standpoint, any single behavioral measure or outcome (e.g., working memory performance) is typically correlated with a range of other behavioral aspects (e.g., personality, cognitive control, etc.), hence reflecting only a very partial aspect of the behavioral phenotype when taken in isolation. To capture the large spectrum of the behavioral phenotypes (i.e., a global behavioral pattern spanning cognition, emotion, and socio‐affective aspects) associated with brain structural features, such as here hippocampal subregional and brain grey matter volume, multivariate approaches are required (Genon, Eickhoff, and Kharabian, 2022; Nicolaisen‐Sobesky et al. 2022). Third, any type of association between brain markers and behavioral markers (be it bivariate or multivariate) found in a sample/subsample is highly likely to result in a replicability failure when investigated in another sample/subsample. Accordingly, a cross‐validation scheme and a very large sample size are required (Genon, Eickhoff, and Kharabian, 2022). Finally, the pattern of associations between interindividual variability in brain structure and interindividual variability in behavior even when evidenced in a large sample often fails to replicate when investigated in a different cohort. In such cases, these association patterns can hardly be considered as reflecting general principles of brain–behavior associations. Evidencing these later requires at least two different cohorts with similar measurements, a requirement that has been particularly difficult to fulfill so far (Genon, Eickhoff, and Kharabian, 2022) and has thus hindered our understanding of associations between the hippocampal–brain structural phenotype and the behavioral phenotype.

Our study addresses these limitations by employing a brain–multiblock multivariate mapping approach (Caplan, McIntosh, and De Rosa 2007; Chiang, Wang, and McKeown 2012) embedded in a machine learning framework to identify replicable multivariate patterns of covariance between different hippocampal subregion structures, whole brain structure, and behavioral phenotypes in population cohorts. Unlike previous studies, this approach allows us to consider the covariance among multiple hippocampal subregions and other brain regions in relation to diverse behavioral variables. This holistic approach allows us to identify and summarize complex patterns of hippocampal–brain–behavior relationships in a two‐dimensional latent space. Therefore, this approach does not consider hippocampal subregions only in relation to behavioral phenotype, but it captures patterns of hippocampal–brain morphological covariance that in turn relates to behavioral phenotypes. By implementing a cross‐cohort discovery and validation framework using two independent cohorts: HCP‐YA cohort and HCP‐A cohort, we could further assess the replicability and generalizability of hippocampal–brain–behavior phenotypes across different age groups in the population. This contrasts with past studies that often suffer from replicability issues due to smaller sample sizes and less comprehensive methodologies. Moreover, we here included a broad spectrum of behavioral variables, spanning different cognitive functions or domains (executive functions, working memory, language, fluid intelligence, …) and socio‐affective aspects (emotion, affects, well‐being, social relationships, stress, …) to capture a more complete picture of the behavioral phenotypes associated with hippocampal morphology. This comprehensive approach allows us to uncover specific hippocampal subregion–brain–behavior patterns that might be obscured in studies with a narrower focus.

## 2 Methods and Materials

### 2.1 Dataset

We employed two large cohorts from the Human Connectome Project (HCP): the HCP Young Adult (HCP‐YA, S1200 release (Van Essen et al. 2013)) and the HCP in Aging (HCP‐A, 2.0 release (Bookheimer et al. 2019)). While the HCP‐YA cohort serves as a comprehensive resource for investigating brain–behavioral patterns in healthy young adults, offering a diverse array of behavioral measurements, the HCP‐A cohort, was chosen for its alignment with the HCP‐YA in behavioral assessments and neuroimaging protocols, but spanning a broader age range than the HCP‐YA (Harms et al. 2018). The common behavioral measures across both cohorts offer the unique opportunity to investigate the replicability of the brain–behavior latent dimensions.

### 2.2 Participants

#### 2.2.1 Human Connectome Project Young Adult

The HCP‐YA cohort comprises neuroimaging and behavioral data from 1206 participants aged 22–37. Derived from 457 families, including twins, and non‐twins, the final sample of 1047 participants (560 females, mean age = [28.78 ± 3.67] years) underwent thorough data quality examinations for structural scans, processing errors, and incomplete data (Table 1).

**TABLE 1:** Demographic characteristics of the used adult cohorts.

	HCP‐YA	HCP‐A
	(Van Essen et al. 2013)	(Bookheimer et al. 2019)
Number of subjects (*N*)	1047	601
Age	28.78 ± 3.67	58.5 ± 14.9
Gender (female)	560	353
Behavioral domains	Emotion Cognition Sleep Episodic memory Executive functions Language Processing speed Self‐regulation/impulsivity Working memory Emotion recognition Negative affect Psychological well‐being Social relationships Stress/self‐efficacy	
Confounding variables	Age, Age^2^, and gender	

#### 2.2.2 Human Connectome Project in Aging

The HCP‐A cohort includes 725 unrelated healthy adults aged 36–100. After exclusions of some participants for technical issues, processing errors, and incomplete behavioral data, the final sample consists of 601 participants (353 females, mean age = [58.5 ± 14.9] years; Table 1).

### 2.3 Behavioral Data

Both cohorts underwent behavioral assessments covering emotion, cognition, sleep, episodic memory, executive functions, language, processing speed, self‐regulation/impulsivity, working memory, emotion recognition, negative affect, psychological well‐being, social relationships, and stress/self‐efficacy. The 32 selected behavioral variables, free of missing values and shared across cohorts, are detailed in (Table S1). Emotion recognition reaction time values (variable ER40_CRT) were uniformly adjusted for interpretability.

### 2.4 Neuroimaging Data Acquisition

The HCP‐YA cohort's neuroimaging data were obtained using a customized 3 T Magnetic Resonance Siemens Skyra “Connectom” scanner at Washington University in St. Louis, United States (Van Essen et al. 2013; Elam et al. 2021). T1‐weighted images utilized a 3D MPRAGE sequence (TR = 2400 ms; TE = 2.14 ms; TI = 1000 ms; voxel size = 0.7 mm isotropic).

In the HCP‐A cohort, neuroimaging data were acquired on standard Siemens 3 T Prisma scanners at four US sites: Washington University in St. Louis, University of California‐Los Angeles, University of Minnesota, and Massachusetts General Hospital (Harms et al. 2018). Common neuroimaging protocols were applied across sites (Bookheimer et al. 2019), with T1‐weighted images acquired using multi‐echo MPRAGE sequences (TR = 2500 ms; TI = 1000 ms; TE = 1.8/3.6/5.4/7.2 ms; voxel size = 0.8 mm isotropic).

### 2.5 Neuroimaging Data Preprocessing

Both cohorts' T1‐w anatomical images underwent processing with the Computational Anatomy Toolbox version 12.5 to compute estimates of grey matter volume (Gaser CaK 2021). After normalization and segmentation steps, grey matter segments were modulated for non‐linear transformations and smoothed. Subsequently, they were parcellated using the Schaefer atlas for 200 cortical regions (Schaefer et al. 2018) and the Melbourne subcortex atlas for 32 subcortical regions (Tian et al. 2020). Overlapping voxels between the subcortical atlas and the cortical atlas were nullified in the cortical atlas to prevent artificial correlations.

### 2.6 Hippocampal Subregions

**FIGURE 1:**
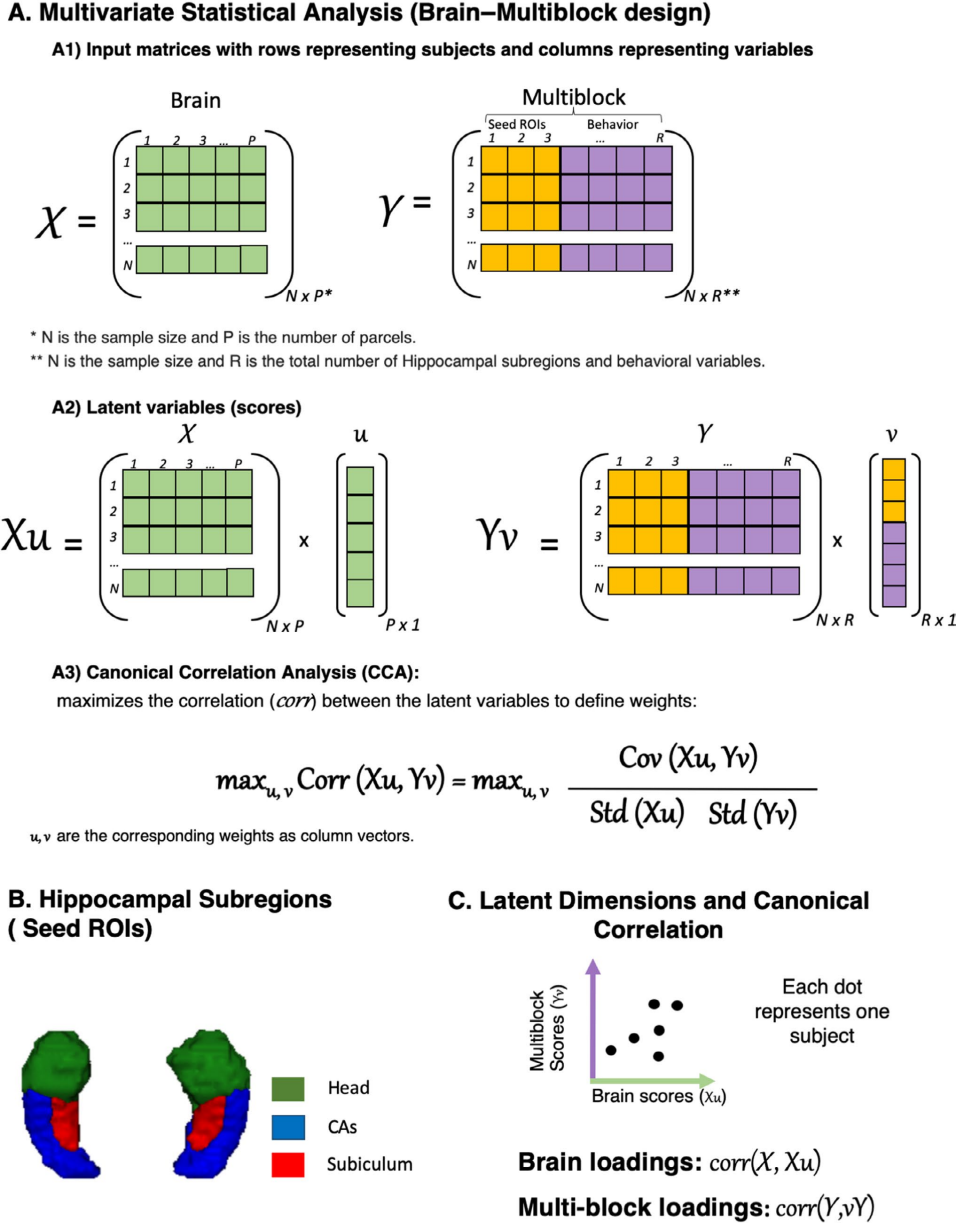
Overview of the brain–multiblock design and analysis framework. Matrix *X* represents parcellated whole‐brain grey matter volumes (except parcels related to bilateral Hippocampus) with dimensions *N* × *P*, where *N* is the sample size and *P* is the number of whole‐brain parcels. Matrix *Y* with dimensions *N* × *R* represents the concatenation of hippocampal subregions (B) and behavioral variables, where *N* is the sample size and *R* is the total number of hippocampal subregions and behavioral variables. Canonical correlation analysis is utilized to identify brain weights (*u*) and multiblock weights (*v*), representing linear combinations of variables in matrices *X* and *Y*, respectively. Projecting the original data *X* and *Y* onto these weights yields scores (Xu and Yv). The model optimizes weights to maximize canonical correlation (i.e., effect size), typically reported with Pearson's correlation between brain scores and multiblock scores. This correlation is depicted as a latent dimension, with each point representing one participant. Variable loadings are derived based on the correlation between the (original) variables and the canonical variate. Loadings here hence reflect the correlation between original variables in *X* and *Y* and brain and multiblock scores, respectively. The figure highlights how latent dimensions are extracted and interpreted.

Hippocampal subregional volumes in young, middle‐aged, and elderly populations were delineated in our prior research (Plachti et al. 2020) using a data‐driven parcellation approach based on the structural covariance pattern across structural MRI data of six large cohorts (*n* = 2594). The subregions were hence delineated based on their distinct brain co‐morphology pattern (resulting from co‐plasticity, coordinated maturation, etc.). This previous work revealed a stable tripartite subdivision which highlighted a very similar pattern for the young, middle‐aged, and elderly group, suggesting a stable pattern of hippocampal differentiation based on brain grey matter covariance across the life span. In the current study, we used the three‐cluster solution derived from young adult cohorts which is openly available on ANIMA (https://anima.fz‐juelich.de/studies/Plachti_DementiaHippocampus_2020; (Reid et al. 2016)) (Figure 1). In this previous parcellation, we highlighted that, in the healthy brain, the hippocampal macrostructure can be discretely categorized along the anterior–posterior axis into an anterior (head) subregion and two posterior (body and tail) subregions (one lateral and one medial subregions). These two latter subregions (posterior lateral and posterior medial) mirror the underlying cytoarchitecture and are accordingly referred to as “posterior CA” and “posterior subiculum” subregions, respectively. Overlapping parcels between the subcortical atlas and the hippocampal volume including hippocampus‐related parcels were nullified in the subcortical atlas to prevent artificial covariance patterns between hippocampal seeds and brain parcels.

### 2.7 Multivariate Statistical Analysis

In this study, we were interested in linking three matrices: subregional hippocampal grey matter volume (seed data), brain parcel grey matter volumes, and behavioral variables. As mentioned earlier, to avoid spurious covariance patterns, hippocampus‐related parcels were excluded from the brain parcel set. Examining the relationships between these three matrices requires a multivariate approach. Partial least squares correlation (PLS) methodology (Krishnan et al. 2011) and Canonical Correlation Analysis (CCA) techniques (Mihalik et al. 2022) represent advanced multivariate statistical tools tailored for exploring the relationship between brain markers/features (such as regional grey matter volumes) and other features or measurements (such as behavioral outcomes). Such multivariate approaches can also be used to explore multivariate covariance between features in one part of the brain (e.g., regional grey matter within hippocampal voxels or subregions) and features in other parts of the brain (e.g., grey matter volume in the cortex). This latter approach is known as a “seed‐PLS”’ approach (Krishnan et al. 2011; Guo et al. 2020; Nordin et al. 2018). While PLS and CCA are typically used for joint analysis of two matrices (i.e., brain features and behavioral measurements), our study extends this approach to accommodate three matrices, highlighting the complexity of our analysis. For this purpose, we implemented a brain–multiblock design (Caplan, McIntosh, and De Rosa 2007; Chiang, Wang, and McKeown 2012) aiming to explore relationships between subregional hippocampal grey matter volume (seed data), whole‐brain parcel grey matter volumes, and behavioral variables. The brain–multiblock design provides a comprehensive framework for exploring complex relationships.

In this study, the brain–multiblock design, where brain–behavior and brain–seed analysis were combined together, converged to reveal latent dimensions (Figure 1). These latent variables capture the optimal relationship between the structural covariance pattern of seed regions and other brain regions across participants. Simultaneously, they assess how these grey matter patterns covary with behavioral outcomes. In each latent dimension, each participant has a brain score which is calculated to measure how strongly the covariance pattern is expressed in that particular participant. Meanwhile, each voxel/subregion has a positive or negative loading that represents its contribution to the pattern described by the latent dimension. Besides, in each latent dimension, every participant has a multiblock score which is calculated to measure how strongly these covariance patterns are related to behavior in that particular participant. Meanwhile, each multiblock variable has a positive or negative loading that represents its contribution to the pattern described by the latent dimension. By extracting latent dimensions, the variability that exists in the population in subregional hippocampal–brain–behavior patterns can be represented in a two‐dimensional space in which the position of any individual represents the extent to which they express a given subregional hippocampal–brain–behavior pattern (Figure 1).

### 2.8 Discovery–Validation Framework

**FIGURE 2:**
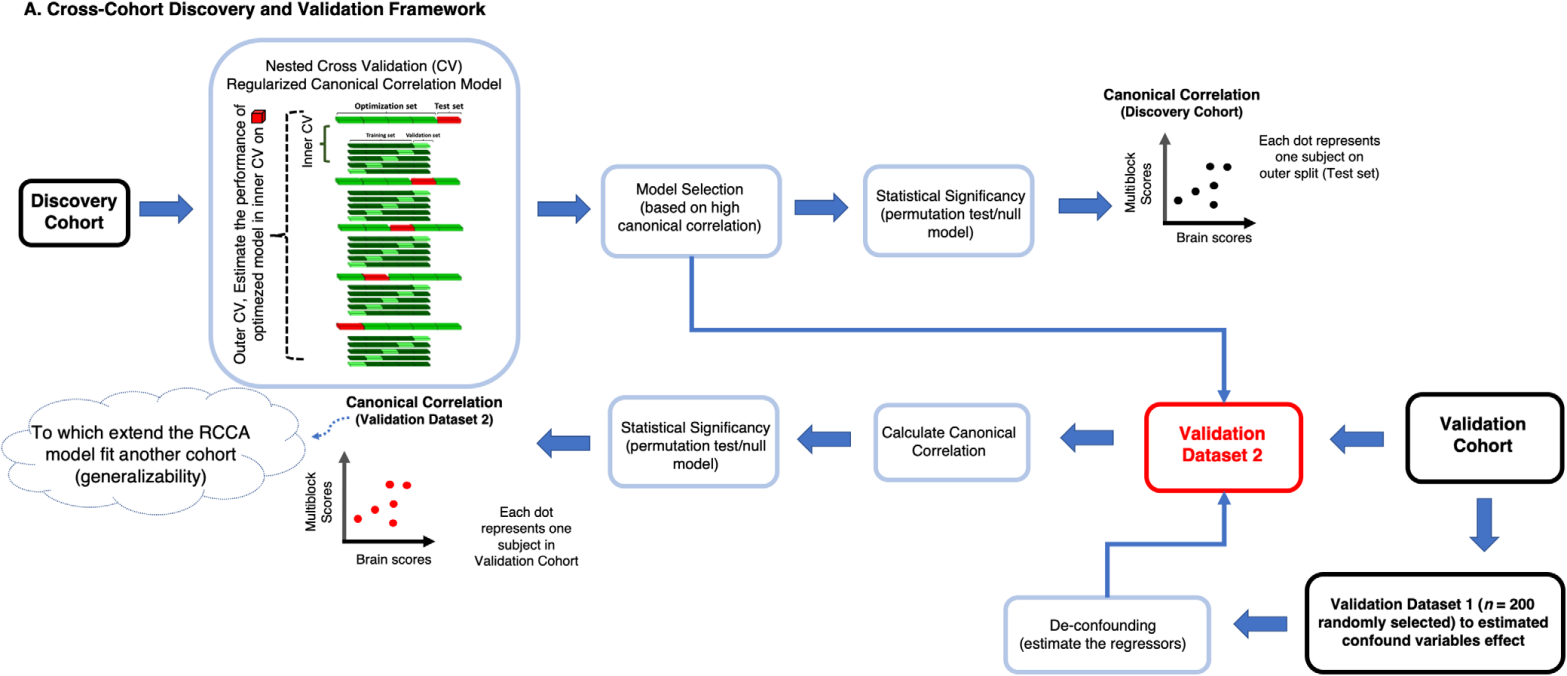
Cross‐Cohort Discovery and Validation Framework. This framework used two independent cohorts: HCP‐YA cohort and HCP‐A cohort as Discovery and Validation cohorts interchangeably to ensure the robustness and generalizability of the findings. Regularized canonical correlation analysis (RCCA) models were trained and evaluated on the discovery cohort using a machine learning framework with nested cross‐validation, consisting of five outer splits and five inner splits. In the outer splits, the data is partitioned into optimization and test sets, while within the inner splits, the optimization set is further divided into training and validation sets. This meticulous division aims to enhance the precision of the model through hyperparameter tuning, model selection, and statistical evaluation. After selecting the best model based on highly significant canonical correlation, the canonical weights (computed in the discovery phase) were used to project the data in the validation onto the identified latent dimensions. This allowed us to assess whether the relationships identified in the discovery cohort between variables could be replicated in the validation cohort.

To ensure the robustness and generalizability of the findings, we implemented a discovery–validation framework using two independent cohorts: HCP‐YA cohort and HCP‐A cohort (Figure 2). In the discovery phase, we identified the relations between interindividual variability in subregional hippocampal networks and variability in behavioral phenotype within the discovery cohort by developing a CCA model. Then, in the validation phase, these findings were tested on an independent cohort (validation cohort) to confirm their generalizability, without adjusting the model or parameters.

### 2.9 Primary Analysis: Discovery Phase

In the primary analysis, we selected the HCP‐YA cohort as the discovery cohort for identifying associations between subregional hippocampal grey matter volume (seed data), brain parcel grey matter volumes, and behavioral variables. To identify latent dimensions within a brain–multiblock design, we applied CCA to three sets of features: regional hippocampal grey matter volume (seed data), brain parcel grey matter volumes, and behavioral variables.

### 2.10 Canonical Correlation Analysis

The canonical correlation analysis (CCA) model received inputs in the form of a brain matrix denoted as *X* and a multiblock matrix represented as *Y*, comprising a concatenation of seed data and behavioral data (Figure 1). CCA identifies brain weights (*u*) and multiblock weights (*v*), which describe linear combinations of the variables in *X* and *Y*, respectively (Krishnan et al. 2011; Mihalik et al. 2022). These weights can be interpreted as a quantification of how much each original variable contributes to the latent dimension (Krishnan et al. 2011; Mihalik et al. 2022). This model selects the weights to maximize the canonical correlation, which corresponds to the correlation of the brain scores (Xu) with the multiblock scores (Yv) (Krishnan et al. 2011; Mihalik et al. 2022). As mentioned earlier, these scores can be interpreted as a quantification of how the latent dimension is expressed in each participant. Notably, in this study, we specifically implemented regularized CCA (RCCA) to counteract overfitting, employing L2‐norm constraints and controlling regularization parameters (cx and cy). The RCCA models were trained and evaluated using a machine learning framework with nested cross‐validation, consisting of 5 outer splits and 5 inner splits (Figure 2). In the outer splits, the data is partitioned into optimization and test sets, while within the inner splits, the optimization set is further divided into training and validation sets. This meticulous division aims to enhance the precision of the model through hyperparameter tuning, model selection, and statistical evaluation. During the cross‐validation process, age, age‐squared, gender, and total intracranial volume (TIV) as a proxy for brain size were regressed out from both brain and multiblock variables to enhance the robustness of the model in a fashion avoiding leakage between the training and test sets (i.e., procedures for de‐confounding the data were estimated on the training set and applied to the validation and test sets). In the case of HCP‐YA, the splitting was done respecting the family structure. Within the nested cross‐validation procedure, performed through the inner splits (optimization sets), RCCA models with varying regularization parameters were fitted to the training sets. The combination that yielded the highest test canonical correlation and stability was then selected as the best model. Moving to the outer splits (test sets), the generalizability of the model was assessed. Here, we focused exclusively on latent dimensions that show significant correlations in all outer splits. The significance of the latent dimensions was assessed as described in the next section. To interpret the significant latent dimensions, we computed and visualized loadings. Brain loadings are derived by correlating the original brain variables (*X*) with the brain scores (Xu). Similarly, multiblock loadings are computed by correlating the original multiblock variables (*Y*) with the multiblock scores (Yv). These loadings reveal which brain and multiblock variables are more strongly associated with the latent dimension. In CCA analysis, multiple latent dimensions can be sequentially extracted from the data, allowing us to distinguish between general and specific patterns of hippocampal–brain–behavior relationships. The first latent dimension typically captures a general pattern of covariance between hippocampus grey matter volume–brain grey matter volume and behavior. Such global pattern is generally driven by broad factors (e.g., some individuals have generally higher grey matter volume across the brain and better cognitive performance), accordingly, they are rarely of interest for disentangling brain–behavior relationships, and they obfuscate the identification of specific hippocampal subregion–brain–behavior associations patterns. Upon identifying a significant latent dimension, its variance was systematically removed from the data through a deflation process, as outlined by (Mihalik et al. 2022). Subsequently, to this deflation step, we sought another latent dimension. The approach used here allows us to investigate the more specific hippocampal subregion–brain–behavior association patterns after removing the variance explained by the first (non‐informative) latent dimension. The significance of the latent dimensions was evaluated using the procedures detailed in the following section.

### 2.11 Statistical Evaluation of Latent Dimensions in the Discovery Phase

The significance of latent dimensions was determined through permutation tests involving 1000 iterations, wherein rows (i.e., the order of the subjects) of the *Y* matrix were shuffled to remove the associations between *X* and *Y* data. For HCP‐YA, the shuffling considered the family structure of the data. We then fit the RCCA models with the optimal regularization parameter (obtained from the original data [i.e., unpermuted]) and projected the permuted test set onto the resulting weight vectors, and the canonical correlation was calculated. *p* values were computed as the percentage of iterations where the canonical correlations obtained from the permuted test set were as extreme as or more extreme than the original canonical correlation derived from the original test set. Multiple statistical testing was accounted for by using Bonferroni correction for multiple comparisons (5 outer splits).

### 2.12 Out‐of‐Cohort Validation Phase in Primary Analysis: Cross‐Cohort Replicability and Generalizability of Latent Dimensions

To ensure that the latent dimensions identified in the discovery phase were not cohort‐specific, we validated the findings by projecting the brain and multiblock variables from the validation cohort (HCP‐A) onto the identified latent dimension in the discovery cohort by multiplying them with the multiblock and brain canonical weights estimated in HCP‐YA (Figure 2). Hence, we tested the generalizability of the latent dimensions across an older adult population (HCP‐A) by assessing to which extent the model identified in the younger cohort fits the data of an older cohort. This projection allowed for the calculation of canonical correlations in the validation cohort using the latent space obtained from the discovery phase. The canonical correlation serves here as an effect size: the greater the magnitude of the canonical correlation, the better the fit of the latent space to the new cohort. As the age range of two cohorts is different, for de‐confounding we could not use the de‐confounding regressors that were estimated on the training set within the discovery cohort. So, we randomly selected 200 subjects from the validation cohort (HCP‐A; validation dataset 1) to estimate the confound regressors and applied them for de‐confounding to the rest of the subjects in validation dataset 2 (Figure 2). To assess whether the brain‐loading spatial maps in the validation cohort overlapped with those of the discovery sample, we the two sets of loadings and tested the significance of these correlations using partial correlation adjusted for spatial distribution of grey matter. Finally, to investigate the similarity of the multiblock loading in the validation and discovery cohorts we performed a Pearson correlation analysis.

### 2.13 Statistical Evaluation of Latent Dimensions in the Validation Phase

Similar to what is done in the discovery phase, the significance of the latent dimension was determined through permutation tests involving 1000 iterations, wherein rows of the Y matrix were shuffled independently within the optimization set from the discovery cohort and validation dataset 2 (Figure 2). For HCP‐YA, the shuffling considered the family structure of the data.

We then fit the RCCA models with the optimal regularization parameter (obtained from the original data in the discovery cohort (i.e., unpermuted)) and projected the permuted validation dataset 2 onto the resulting weight vectors, and the canonical correlation was calculated. *p* values were computed as the percentage of iterations where the canonical correlations obtained from the permuted validation dataset 2 were as extreme as or more extreme than the original canonical correlation derived from the original validation dataset 2.

### 2.14 Replication Analysis

To further strengthen the generalizability of the findings, we performed a replication analysis by reversing the roles of the discovery and validation cohorts. This additional step ensured that the relationships identified between subregional hippocampal grey matter volume (seed data), brain parcel grey matter volumes, and behavioral variables were robust across both age groups.

In the replication analysis, the HCP‐A cohort served as the discovery cohort. RCCA was applied to identify latent dimensions between subregional hippocampal grey matter volume (seed data), brain parcel grey matter volumes, and behavioral variables in the older adult population. The canonical weights derived from the HCP‐A cohort were then applied to the HCP‐YA cohort, and canonical correlations were recalculated in the younger cohort using the projected data. This allowed us to assess whether the latent dimensions identified in the older cohort replicated in the younger cohort. Here, we used the same approach for de‐confounding the validation dataset as described in the previous section and Figure 2.

## 3 Results

This study focused on investigating the association between the morphological network of hippocampal subregions and behavior. Utilizing a multiblock design that concatenated seed and behavioral data, we employed hippocampal subregions defined in a prior study (Plachti et al. 2020). In this prior work, the hippocampal macrostructure was delineated into three subregions along the anterior–posterior axes in the healthy brain. Measures of mean grey matter volume from these subregions served as seed data, alongside 32 carefully selected behavioral variables spanning alertness, cognition, and emotion (S1 Table), creating a multiblock cohort. The chosen behavioral variables covered relevant phenotypes, were available in both cohorts (HCP‐YA and HCP‐A), and were devoid of missing data. The set of brain structural features included parcelwise measures of mean grey matter volume across 232 cortical and subcortical parcels. Age, age‐square, gender, and TIV as a proxy for brain size were regressed out from both the brain and multiblock variables in a fashion preventing train‐test leakage. We implemented a cross‐cohort discovery and validation framework for ensuring the robustness and generalizability of the latent dimensions on the designed brain–multiblock. The RCCA was applied to the discovery cohort as embedded in a machine learning framework, incorporating nested cross‐validation for identifying stable latent dimensions.

As mentioned earlier, we focused exclusively on latent dimensions where all outer splits exhibited significant canonical correlation. Thus, we found four significant latent dimensions when using HCP‐YA as the discovery cohort and all identified latent dimensions appeared as generalizable in the older cohort (HCP‐A) as the validation cohort. However, in replication analysis, when HCP‐A was the discovery cohort, we only captured two significant latent dimensions and both of them were generalizable in the younger cohort (HCP‐YA) as the validation cohort. The first two latent dimensions appear as replicable hippocampal–brain–behavior phenotypes across cohorts with different age groups.

In the discovery phase, the first latent dimension in both primary analysis and replication analysis represents the general covariance pattern between the whole hippocampus, the whole brain, and behavioral measures (see Figures S1–S3). Upon deflating this general covariance pattern, we observe hippocampal subregional differentiation in the brain and phenotypical covariance in the subsequent latent dimension (the second latent dimension). For both analyses, this latent dimension highlights a left hippocampus head (vs. all other hippocampal subregions) positive covariance pattern in the brain with a specific range of behavioral variables. Consequently, in the following section, we focus on the second latent dimension, while other latent dimensions are further described in the Supporting Information (see Figures S1–S5 and Tables S2–S3). Our out‐of‐cohort validation phase analyses further enable us to examine the generalizability of latent dimensions when applied to a new cohort independent from the cohort in which it has been discovered/modeled. It is noteworthy that, according to our supplementary analyses, our results do not seem to be influenced by potential spurious effects of the scanning site in the HCP‐A cohort (see Supporting Information for details, Figure S6).

### 3.1 Discovery Phase: Second Latent Dimension in HCP‐YA Cohort

**FIGURE 3:**
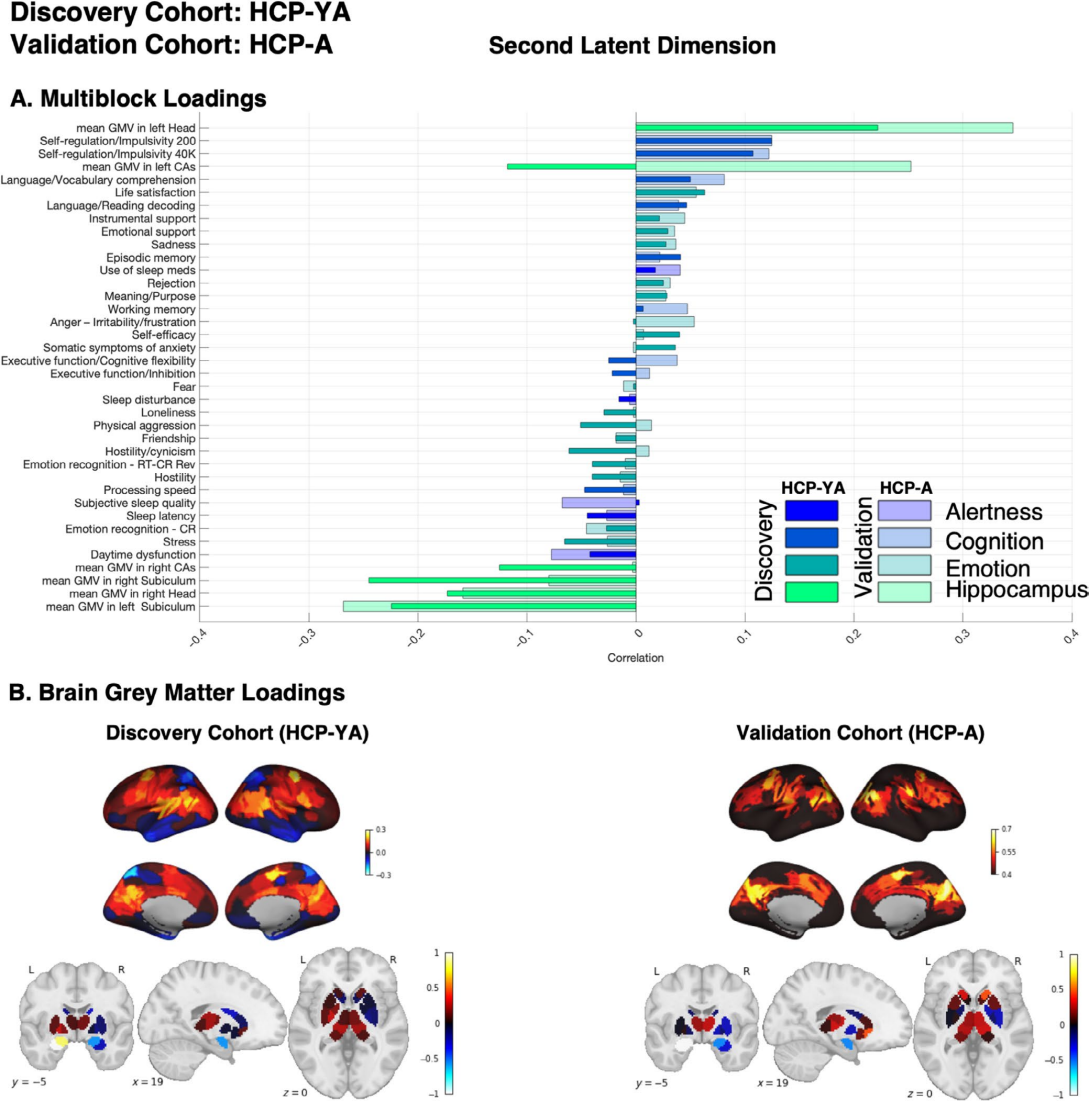
Second latent dimension: Discovery cohort (HCP‐YA) and Validation cohort (HCP‐A). (A) Multiblock loadings; (B) Brain grey matter volume loadings. Both loadings were primarily used for interpreting how variables in the multiblock data and whole brain grey matter volume contributed to the identified second latent dimension A. Dark and light colors represent loadings for the discovery and validation cohorts, respectively. The loadings are calculated for the best model based on high effect size (canonical correlation) across five outer splits. The color‐map bars illustrate multiblock variables associated with various domains such as alertness, cognition, emotion, and hippocampal subregions. (B) Cortical and subcortical patterns of brain loadings are shown separately for visualization purposes. Thresholding was applied in the brain loadings cortical maps purely for visualization purposes to highlight key contributing regions. It was not based on any statistical criteria and does not reflect significance. The subcortical slice corresponds to MNI coordinates: 19, −5, 0. In both cortical and subcortical maps, red indicates positive loadings and blue indicates negative loadings.

In discovery phase of primary analysis, the second latent dimension (Figure 3) highlighted a left hippocampus head positive covariance pattern with grey matter volume in the left amygdala, the bilateral thalamus, the bilateral temporoparietal cortices, the left posterior globus pallidus, the left auditory cortex, the left precuneus and posterior cingulate cortex, the right precuneus, the right retrosplenial, and the right frontal eye‐field. Negative covariance (brain negative loadings) appeared mainly in the right amygdala and the right posterior putamen, caudate, and globus pallidus. In the CCA, this regional–hippocampal–brain covariance pattern demonstrated positive associations at the behavioral level with self‐regulation and efficiency, life satisfaction, language processing, and sustained attention abilities. In contrast, it was negatively correlated with sleep problems, aggressive behavior, stress, hostility/cynicism, attention problems, and cognitive flexibility.

In other words, participants showing relatively higher grey matter volume in the left hippocampus head's (but relatively lower in the right hippocampus head and bilateral hippocampal posterior regions) morphological network also display relatively higher self‐regulation and efficacy, life satisfaction, and sustained attention abilities, along with lower levels of aggressive behavior, stress, hostility/cynicism, and attention problems, but also lower cognitive flexibility. Conversely, participants with relatively higher grey matter volume in the right hippocampus head and more posterior hippocampal regions show higher grey matter volume in the right amygdala and the right posterior putamen, caudate, and globus pallidus. These individuals exhibit lower self‐regulation abilities, higher aggressivity/hostility, and cynicism, but also higher stress and more sleep problems.

### 3.2 Validation Phase: Testing the Latent Dimension in HCP‐A Cohort

**FIGURE 4:**
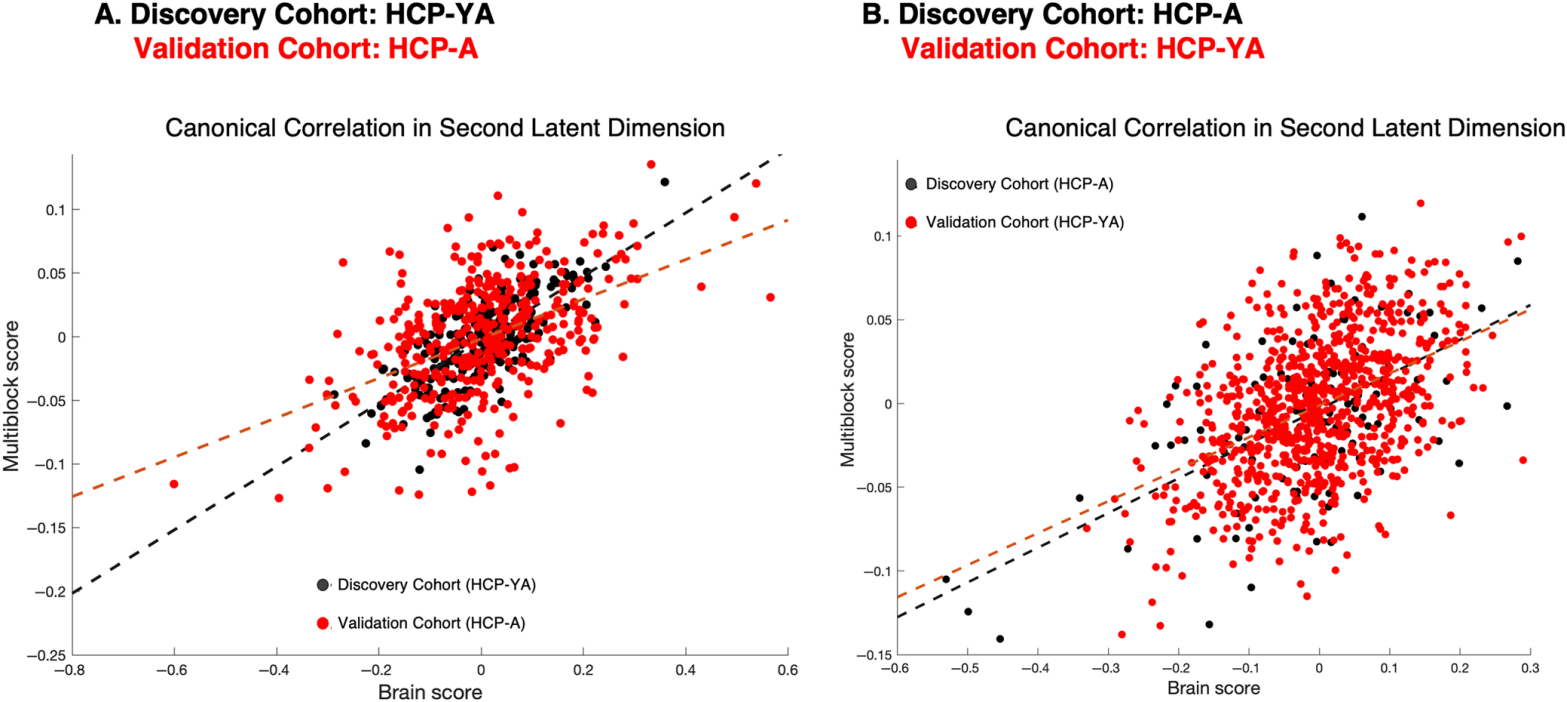
Cross‐cohort replicability and generalizability of the second latent dimension. This figure illustrates the effect sizes of canonical correlations for the second latent dimension in both discovery and validation cohorts. (A) In the primary analysis, using the HCP‐YA as the discovery cohort, the effect size for the significant canonical correlation was 0.72, while the effect size in the validation cohort (HCP‐A) was 0.48. (B) In the replication analysis, where HCP‐A served as the discovery cohort and HCP‐YA as the validation cohort, the effect sizes were 0.58 and 0.45, respectively. All effect sizes are significant, indicating robust relationships between hippocampal–brain–behavior variability across cohorts.

In the validation phase, we tested the replicability and generalizability of identified latent dimensions in HCP‐YA using an independent sample (HCP‐A). In each latent dimension, the weights derived from the HCP‐YA sample were used to estimate canonical correlation and Multiblock and brain loadings in the HCP‐A sample. The magnitude of the canonical correlation indicates the extent to which the model (the modeled latent dimension and thus latent space) fits the data. The significant canonical correlation observed in the validation cohort, when projected onto the weight vectors derived in the discovery phase, served as an indicator of the model's generalizability. In Figure 4A, we showed a canonical correlation of the second latent dimension (i.e., effect sizes) in both discovery and validation cohorts. In the primary analysis, the effect size of the significant canonical correlation for the second latent dimension in the discovery cohort (HCP‐YA) was 0.72 and in the validation cohort (HCP‐A) was 0.48. Comparing their loadings, we found strong positive correlations between the two samples (*r* = 0.69, *p*_corr_ < 0.001 and *r* = 0.70, *p*_corr_ < 0.001 for multiblock and brain, respectively; see Figure 3B for brain loading the correlation adjusted for the auto‐spatial correlation of the brain data). In the validation cohort (HCP‐A) (Figure 3), the left hippocampus head also showed a positive covariance pattern with grey matter volume in the left amygdala and a negative covariance pattern with the right amygdala, bilateral posterior caudate, and right globus pallidus. However, the left hippocampus head in the aging cohort highlighted positive covariance with overall the bilateral cerebral cortex, but that was the highest in the cortical regions highlighted in the HCP‐YA including the bilateral temporo‐parietal, the left auditory cortex, the left precuneus, and the posterior cingulate cortex, the right precuneus, the right retrosplenial, and the right frontal eye‐field. This analysis revealed that projecting HCP‐A data to the second latent dimension identified by HCP‐YA preserves the multivariate patterns of covariance between the left hippocampus head, whole brain structure, and behavioral phenotypes. As the measured behavioral and imaging data were similar between the two cohorts, this out‐of‐cohort validation constitutes a test of the true validity of the brain–behavior relationship.

### 3.3 Replication by Swapping Discovery and Validation Cohorts

**FIGURE 5:**
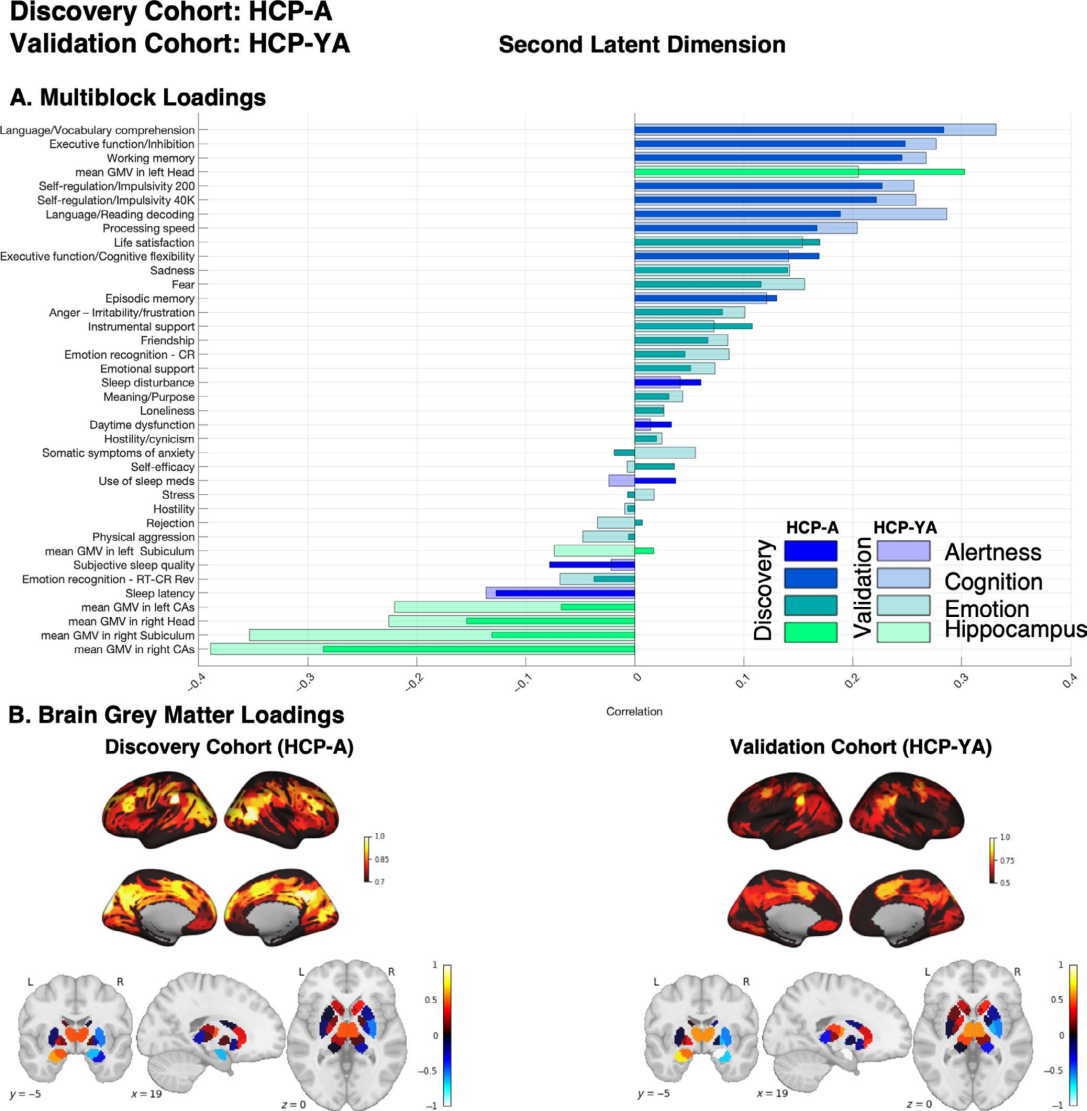
Second latent dimension in replication analysis: Discovery cohort (HCP‐A) and Validation cohort (HCP‐YA). (A) Multiblock loadings; (B) Brain grey matter volume loadings. Both loadings were primarily used for interpreting how variables in the multiblock data and whole brain grey matter volume contributed to the identified second latent dimension A. Dark and light colors represent loadings for the discovery and validation cohorts, respectively. The loadings are calculated for the best model based on high effect size (canonical correlation) across five outer splits. The color‐map bars illustrate multiblock variables associated with various domains such as alertness, cognition, emotion, and hippocampal subregions. (B) Cortical and subcortical patterns of brain loadings are shown separately for visualization purposes. Thresholding was applied in the brain loadings cortical maps purely for visualization purposes to highlight key contributing regions. It was not based on any statistical criteria and does not reflect significance. The subcortical slice corresponds to MNI coordinates: 19, −5, 0. In both cortical and subcortical maps, red indicates positive loadings and blue indicates negative loadings.

When the mapping between hippocampal subregions, other brain regions, and behavioral variables was explored in the HCP‐A as a discovery cohort, a similar latent dimension (as found in the HCP‐Y) was found (Figure 5) showing a left hippocampus head (vs. right hippocampal head and posterior subregions) positive covariance pattern with grey matter volume in the left amygdala and overall the bilateral cerebral cortex, but that was the highest in the dorsal/lateral prefrontal cortex, the middle and posterior cingulate cortex/precuneus, Intraparietal sulcus, inferior parietal lobule, and superior/parieto‐occipital cortex. Negative covariance (brain negative loadings) appeared mainly in the right amygdala and right putamen, and right posterior globus pallidus. In the CCA, this regional–hippocampal–brain covariance pattern was positively associated at the behavioral level with language processing, working memory, executive functions, self‐regulation, life satisfaction, self‐efficacy, processing speed, episodic memory, and the use of instrumental support. In contrast, it was negatively correlated with sleep latency and poor sleep quality.

In other words, older participants with relatively higher grey matter volume in the left hippocampus head (but relatively lower in the right hippocampus head and Cornu Ammonis (CAs) areas) tend to exhibit higher grey matter volume in the cerebral cortex. Additionally, they display relatively higher short‐ and long‐term memory abilities, self‐regulation and efficacy, life satisfaction, and higher executive functions as well as better sleep quality. However, they also show a tendency toward more sadness. Conversely, older participants with relatively higher grey matter volume in the right hippocampus and CAs regions tend to exhibit higher grey matter volume in the right amygdala and putamen and display lower self‐regulation and efficacy, lower sleep quality, but also lower sadness.

In the validation phase, we tested the replicability and generalizability of identified latent dimensions in HCP‐A using the HCP‐YA as a validation cohort. Similar to primary analysis, in each latent dimension, the weights derived from the HCP‐A sample were used to estimate canonical correlation and multiblock and brain loadings in the HCP‐YA cohort. In the replication analysis, the effect size of the second latent dimension in the discovery cohort (HCP‐A) was 0.58 and in the validation cohort (HCP‐YA) was 0.45 (Figure 3B). Comparing their loadings, we found strong positive correlations between the two cohorts (*r* = 0.94, *p*_corr_ < 0.001 and *r* = 0.89, *p*_corr_ < 0.001 for multiblock and brain, respectively; see Figure 5 for brain loading the correlation adjusted for the auto‐spatial correlation of the brain data). In this validation cohort (HCP‐YA), both multiblock and brain loadings were very similar to those found in the discovery cohort (HCP‐A). This analysis revealed that the relationships identified between the grey matter volume of the left hippocampus head, specific cortical and subcortical brain patterns, and behavioral variables including self‐regulation and efficacy, sleep quality, and cognitive ability were overall robust across both age groups.

## 4 Discussion

### 4.1 A Stable and Replicable Latent Dimension Highlighting the Left Anterior Hippocampus

In this study, we used a data‐driven approach across two distinct cohorts to examine how interindividual variability in subregional hippocampal grey matter volume relates to interindividual variability in brain grey matter and to interindividual variability in behavioral measures conjointly. Strikingly, we identified a stable, latent dimension associated with the left anterior hippocampus' head grey matter network, encompassing regions such as the left amygdala and posterior medial cortex. Notably, the model capturing variability in the discovery cohort also accounted for variability in the validation cohort, demonstrating its generalizability. This consistency across independent cohorts suggests that this latent dimension represents a fundamental, robust structure of hippocampal–brain co‐morphology and its association with behavioral traits across the population. Several aspects can be noted regarding this latent dimension. First, interindividual variability in the anterior (head) versus posterior subregions appears as different poles of variability in the population, but more specifically, the left hippocampus' head structure appears as loading in a different direction than its right homolog. Second, at the brain level, the left anterior hippocampus' head appears particularly morphologically coupled with the left amygdala, a region engaged in emotion processing, but also with cortical regions, such as the median posterior cortical subregions that support high‐level, integrative cognitive processes. Finally, higher grey matter volume in this network in the healthy population relates to a behavioral phenotype characterized by higher self‐regulation, but which could also imply lower performance on some specific tests. All these findings are further discussed below.

### 4.2 Left (vs. Right) Anterior Hippocampal Brain Morphology Network

Our data‐driven approach highlights a left hippocampal head grey matter network as a stable dimension of interindividual variability. Interestingly, the left hippocampus appears specifically on one pole of the dimension while the right hippocampus' head loads on the other pole of the dimension. This result highlights the importance of considering the structural and functional hippocampal asymmetry to better understand behavioral phenotypes in the healthy and diseased population (Genon et al. 2021). Due to the relative weight of animal models, in particular rodent models, in the study of hippocampal function and pathologies and the lack of consideration of left–right asymmetry in these models, our understanding of hippocampal structural and functional asymmetry and its implication in human behavior and brain diseases (such as Alzheimer's disease, schizophrenia, epilepsy, and anxio‐depressive disorders) remain relatively limited (Nemati et al. 2023). Yet, the examination of hippocampal morphometry in humans has suggested volumetric differences between the right and the left hemispheres that may be specific to the anterior part of the hippocampus (Woolard and Heckers 2012). Furthermore, a right–left global hippocampal morphological asymmetry has been often reported in the normal aging population and this asymmetry or the right/left ratio has been generally found to be altered in AD (Geroldi et al. 2000; Barnes et al. 2005; Jahanshahi, Naghdi Sadeh, and Khezerloo 2023; Poloni et al. 2021). However, in this latter literature, a common specific asymmetry pattern across studies does not appear, possibly because the whole hippocampus was examined without considering anterior–posterior differentiation. The relevance of this subregional differentiation in line with our results is further discussed in the next section.

At the functional level, the left hemisphere dominance for linguistic functions is generally well known and for the hippocampus, a left dominance for episodic and contextual memory is generally put in contrast to a right dominance for spatial navigation (Nemati et al. 2023). However, beyond these very general functional lateralization hypotheses, finer specifications and implications thereof for explaining variability in behavioral phenotype are missing. Yet, these specifications would contribute to better understand clinical profiles including clinical heterogeneity. For example, hemispheric and anterior–posterior asymmetry have been reported to be significantly more marked in semantic dementia atrophy than in AD patients with greater involvement of the left and anterior hippocampal subfields in the former (La Joie et al. 2013). In this respect, our study indirectly suggests that individuals with lower grey matter volume relatively specifically in the left hippocampus' head and the left amygdala might show lower self‐regulation profile and lower performance at tasks requiring higher cognitive control, such as working memory, inhibition, and language/vocabulary comprehension hence providing more specific expectations on cognitive alterations that may be expected in clinical populations in which the left anterior hippocampal morphological network is affected. In the next section, we further discuss the relevance of anterior vs. posterior hippocampal morphological networks in relationship to behavioral phenotype.

### 4.3 Left Anterior (vs. Posterior) Hippocampal Brain Morphology Network and Self‐Regulation

Interestingly, our dimensional approach also identifies more specifically the anterior part of the hippocampus as relating to this self‐regulation phenotype (in contrast to the posterior parts of the hippocampus). In that framework, our previous study has suggested that the hippocampus' head volume may co‐maturate with most of the cortical sheet in childhood hence participating in the early development of key neurocognitive functioning (Plachti et al. 2023), in particular executive functions and high‐level conceptual/language skills. Accordingly, cortical structural variability in healthy adulthood may covary more specifically with anterior hippocampal structural variability than with posterior hippocampal variability (Woolard and Heckers 2012). Yet, at the functional level, the anterior hippocampus is known to be more engaged in self‐related processes, working closely in interaction with the amygdala to process internal information (Genon et al. 2021; Plachti et al. 2019; Maleki Balajoo et al. 2023). In that framework, our results suggest that individuals with more grey matter volume in the anterior hippocampal–cortical network show better self‐regulation associated with higher self‐efficacy and life satisfaction. Given the implication in life of such a brain–behavior phenotype in adulthood, future studies should elucidate how this morphological coupling takes place across development (in particular in childhood) and what the exposome and genetic factors that influence this left anterior hippocampal network development.

### 4.4 The Left Anterior Hippocampal–Brain Morphologic Networks: The Two Sides of the Coin

Our dimensional approach highlights a left anterior hippocampal–brain morphologic network whose morphological development is associated with self‐regulation abilities and performing relatively better in many standard psychometric tests. Nevertheless, the multivariate mapping used here also suggests that this phenotype could go along with higher cognitive rigidity (lower cognitive flexibility) and possibly more sadness in older populations. This pattern of results highlights the complexity of the associations between grey matter volume and behavioral phenotype in the population beyond the traditionally assumed “the bigger the better” hypothesis (Genon, Eickhoff, and Kharabian, 2022). In particular, in anxio‐depressive phenotype, a higher volume of the amygdala has been frequently reported (Oyarce et al. 2020; Besteher, Gaser, and Nenadić 2020). Nevertheless, subregional hippocampal patterns were rarely investigated hindering our understanding of the amygdala–hippocampus morphological coupling in aspects of the behavioral phenotype that may extend to dysfunction in subclinical and clinical populations, such as anxio‐depressive disorders.

In our dimension approach, grey matter volume pattern within the left anterior hippocampal–brain morphologic networks is put in contrast (on the opposite pole) to grey matter volume pattern in the right posterior hippocampal and right subcortical structures. According to the latent space, individuals who show relatively higher grey matter volume within this later morphological network would show higher cynicism, higher hostility, higher sleep latency, and higher stress. Thus, we could speculate that higher left anterior–hippocampal–cortical morphology goes along with better cognitive control and emotional regulation that participate in performance in standard cognitive paradigms, but at the expense of right subcortical volume that is associated in some cases with negative behavioral aspects.

In other words, generally, we here speculate that the relative expression of a subregional hippocampal–brain pattern relates to the relative expression of a behavioral phenotype. More concretely, the structural pattern of left versus right and anterior versus posterior hippocampus would be relevant to explain the individual behavioral phenotype. In this study, we focused specifically on healthy adult populations, and this leads to the promotion of a left anterior hippocampo‐cortical network going along with several positive behavioral aspects. However, more extreme structural expression of this pattern might be related to lower behavioral performance and to over self‐regulation leading to cognitive rigidity. Nevertheless, evidencing these extreme brain‐behavior phenotypes would require extended behavioral phenotyping in clinical populations in which the hippocampus is typically affected, such as anxio‐depressive disorders, sleep disorders, and autoimmune limbic encephalitis and/or temporal lobe epilepsy.

Generally, the multivariate framework linking hippocampal networks to behavioral phenotypes in the current study can be used to investigate how alterations of these networks relate to behavioral symptoms in clinical populations. This insight would be particularly useful in disorders in which patients show either lower or higher self‐regulation together with hippocampal alterations, such as autism spectrum disorder (Banker et al. 2021), fetal alcohol spectrum disorders (Popova et al. 2023), and anxiety disorders (Lipschutz et al. 2024). Ultimately, future studies should contribute to elucidate how the structural and functional maturation of the left anterior hippocampal networks during childhood play a role in self‐regulation disorders.

## 5 Conclusions and Perspectives

In sum, for the first time in this study, we used a multiblock multivariate approach to map multivariate associations between grey matter volume in hippocampal subregions, grey matter volume in the brain, and behavioral phenotype. By implementing this approach within a machine learning framework, we were able to identify stable latent dimensions, and through a cross‐cohort discovery and validation framework, we assessed the replicability and generalizability of hippocampal–brain–behavior phenotypes across age groups. We focused on a latent dimension that was both replicable and generalizable across cohorts, suggesting it captures a core aspect of the hippocampal–brain–behavior phenotype in healthy adults. Our findings highlighted a left anterior hippocampal morphological network including the left amygdala and posterior midline cortical structure whose expression relates to higher self‐regulation and better performance at standard neuropsychological tests. Future studies should investigate the structural development of this morphological network across childhood and the genetic and exposome factors influencing it.

Furthermore, future studies should investigate the extreme expression of this morphological network in clinical populations and how the expression of the hippocampal–brain–behavior phenotype in middle‐aged adults relates to neurocognitive alterations in older age, in particular in neurodegenerative diseases. In line with these perspectives, the approach developed in this study is openly available and can be easily applied for future studies to investigate hippocampal–brain–behavior phenotype in developmental and clinical populations.

## Author Contributions

**S.M.B.:** conceptualized the study, developed codes, prepared data, performed analyses, contributed to discussion and interpretation of results, and wrote the first draft. **A.P.** and **D.D.:** contributed to discussion and interpretation of results. **E.N.‐S.:** contributed to the data preparation and code development. **F.H.:** contributed to data preparation and preprocessing. **S.B.E.:** acquired funding, and contributed to discussion and interpretation of results. **S.G.:** acquired funding, conceptualized the study, contributed to discussion and interpretation of results, and wrote the first draft. All authors contributed to the revision of the manuscript.

## Ethics Statement

Ethics protocols for data analyses were approved by the Heinrich Heine University Düsseldorf ethics committee (No. 4039). Informed consents were obtained from HCP participants (Elam et al. 2021).

## Conflicts of Interest

The authors declare no conflicts of interest.

## Supporting information


Data S1.

## Data Availability

Access to data of the HCP can be requested on ConnectomeDB (https://db.humanconnectome.org/app/template/Login.vm). The code used for the machine learning framework has been made publicly available at https://github.com/anaston/cca_pls_toolkit. MATLAB R2020b and python3 were used for data curation; the RCCA analyses and the machine learning framework were implemented in MATLAB R2020b; Computational Anatomy Toolbox version 12.5 was used to estimate grey matter volume.

## References

[hbm70099-bib-0001] Banker, S. M. , X. Gu , D. Schiller , and J. H. Foss‐Feig . 2021. “Hippocampal Contributions to Social and Cognitive Deficits in Autism Spectrum Disorder.” Trends in Neurosciences 44, no. 10: 793–807. 34521563 10.1016/j.tins.2021.08.005 PMC8484056

[hbm70099-bib-0002] Barnes, J. , R. I. Scahill , J. M. Schott , C. Frost , M. N. Rossor , and N. C. Fox . 2005. “Does Alzheimer's Disease Affect Hippocampal Asymmetry? Evidence From a Cross‐Sectional and Longitudinal Volumetric MRI Study.” Dementia and Geriatric Cognitive Disorders 19, no. 5–6: 338–344. 15785035 10.1159/000084560

[hbm70099-bib-0003] Besteher, B. , C. Gaser , and I. Nenadić . 2020. “Brain Structure and Subclinical Symptoms: A Dimensional Perspective of Psychopathology in the Depression and Anxiety Spectrum.” Neuropsychobiology 79, no. 4–5: 270–283. 31340207 10.1159/000501024

[hbm70099-bib-0004] Bookheimer, S. Y. , D. H. Salat , M. Terpstra , et al. 2019. “The Lifespan Human Connectome Project in Aging: An Overview.” NeuroImage 185: 335–348. 30332613 10.1016/j.neuroimage.2018.10.009 PMC6649668

[hbm70099-bib-0005] Caplan, J. B. , A. R. McIntosh , and E. De Rosa . 2007. “Two Distinct Functional Networks for Successful Resolution of Proactive Interference.” Cerebral Cortex 17, no. 7: 1650–1663. 16968868 10.1093/cercor/bhl076

[hbm70099-bib-0006] Chiang, J. , Z. J. Wang , and M. J. McKeown . 2012. “A Multiblock PLS Model of Cortico‐Cortical and Corticomuscular Interactions in Parkinson's Disease.” NeuroImage 63, no. 3: 1498–1509. 22982102 10.1016/j.neuroimage.2012.08.023

[hbm70099-bib-0007] Clark, I. A. , A. M. Monk , V. Hotchin , et al. 2020. “Does Hippocampal Volume Explain Performance Differences on Hippocampal‐Dependant Tasks?” NeuroImage 221: 117211. 32739555 10.1016/j.neuroimage.2020.117211 PMC7762813

[hbm70099-bib-0008] Elam, J. S. , M. F. Glasser , M. P. Harms , et al. 2021. “The Human Connectome Project: A Retrospective.” NeuroImage 244: 118543. 34508893 10.1016/j.neuroimage.2021.118543 PMC9387634

[hbm70099-bib-0009] Gaser CaK, F. 2021. “Manual Computational Anatomy Toolbox‐ cat12.”

[hbm70099-bib-0010] Genon, S. , B. C. Bernhardt , R. La Joie , K. Amunts , and S. B. Eickhoff . 2021. “The Many Dimensions of Human Hippocampal Organization and (Dys)function.” Trends in Neurosciences 44, no. 12: 977–989. 34756460 10.1016/j.tins.2021.10.003 PMC8616840

[hbm70099-bib-0011] Genon, S. , S. B. Eickhoff , and S. Kharabian . 2022. “Linking Interindividual Variability in Brain Structure to Behaviour.” Nature Reviews. Neuroscience 23, no. 5: 307–318. 35365814 10.1038/s41583-022-00584-7

[hbm70099-bib-0012] Genon, S. , A. Reid , R. Langner , K. Amunts , and S. B. Eickhoff . 2018. “How to Characterize the Function of a Brain Region.” Trends in Cognitive Sciences 22, no. 4: 350–364. 29501326 10.1016/j.tics.2018.01.010 PMC7978486

[hbm70099-bib-0013] Geroldi, C. , M. Laakso , C. DeCarli , et al. 2000. “Apolipoprotein E Genotype and Hippocampal Asymmetry in Alzheimer's Disease: A Volumetric MRI Study.” Journal of Neurology, Neurosurgery & Psychiatry 68, no. 1: 93–96. 10601411 10.1136/jnnp.68.1.93 PMC1760588

[hbm70099-bib-0014] Guo, P. , Q. Li , X. Wang , et al. 2020. “Structural Covariance Changes of Anterior and Posterior Hippocampus During Musical Training in Young Adults.” Frontiers in Neuroanatomy 14: 20. 32508600 10.3389/fnana.2020.00020 PMC7248297

[hbm70099-bib-0015] Harms, M. P. , L. H. Somerville , B. M. Ances , et al. 2018. “Extending the Human Connectome Project Across Ages: Imaging Protocols for the Lifespan Development and Aging Projects.” NeuroImage 183: 972–984. 30261308 10.1016/j.neuroimage.2018.09.060 PMC6484842

[hbm70099-bib-0016] Jahanshahi, A. R. , R. Naghdi Sadeh , and D. Khezerloo . 2023. “Atrophy Asymmetry in Hippocampal Subfields in Patients With Alzheimer's Disease and Mild Cognitive Impairment.” Experimental Brain Research 241, no. 2: 495–504. 36593344 10.1007/s00221-022-06543-z

[hbm70099-bib-0017] Kharabian Masouleh, S. , S. B. Eickhoff , F. Hoffstaedter , and S. Genon . 2019. “Alzheimer's Disease Neuroimaging I. Empirical Examination of the Replicability of Associations Between Brain Structure and Psychological Variables.” eLife 8: 43464. 10.7554/eLife.43464 PMC6483597 30864950

[hbm70099-bib-0018] Kharabian Masouleh, S. , S. B. Eickhoff , S. Maleki Balajoo , E. Nicolaisen‐Sobesky , B. Thirion , and S. Genon . 2022. “Empirical Facts From Search for Replicable Associations Between Cortical Thickness and Psychometric Variables in Healthy Adults.” Scientific Reports 12, no. 1: 13286. 35918502 10.1038/s41598-022-17556-7 PMC9345926

[hbm70099-bib-0019] Kharabian Masouleh, S. , A. Plachti , F. Hoffstaedter , S. Eickhoff , and S. Genon . 2020. “Characterizing the Gradients of Structural Covariance in the Human Hippocampus.” NeuroImage 218: 116972. 32454206 10.1016/j.neuroimage.2020.116972

[hbm70099-bib-0020] Krishnan, A. , L. J. Williams , A. R. McIntosh , and H. Abdi . 2011. “Partial Least Squares (PLS) Methods for Neuroimaging: A Tutorial and Review.” NeuroImage 56, no. 2: 455–475. 20656037 10.1016/j.neuroimage.2010.07.034

[hbm70099-bib-0021] La Joie, R. , A. Perrotin , V. de La Sayette , et al. 2013. “Hippocampal Subfield Volumetry in Mild Cognitive Impairment, Alzheimer's Disease and Semantic Dementia.” NeuroImage: Clinical 3: 155–162. 24179859 10.1016/j.nicl.2013.08.007 PMC3791274

[hbm70099-bib-0022] Lipschutz, R. , A. Powers , S. T. Minton , et al. 2024. “Smaller Hippocampal Volume Is Associated With Anxiety Symptoms in High‐Risk Black Youth.” Journal of Mood & Anxiety Disorders 7: 100065. 39391077 10.1016/j.xjmad.2024.100065 PMC11466052

[hbm70099-bib-0023] Maleki Balajoo, S. , S. B. Eickhoff , S. K. Masouleh , et al. 2023. “Hippocampal Metabolic Subregions and Networks: Behavioral, Molecular, and Pathological Aging Profiles.” Alzheimer's & Dementia 19, no. 11: 4787–4804. 10.1002/alz.13056 PMC10698199 37014937

[hbm70099-bib-0024] Mihalik, A. , J. Chapman , R. A. Adams , et al. 2022. “Canonical Correlation Analysis and Partial Least Squares for Identifying Brain‐Behavior Associations: A Tutorial and a Comparative Study.” Biological Psychiatry: Cognitive Neuroscience and Neuroimaging 7, no. 11: 1055–1067. 35952973 10.1016/j.bpsc.2022.07.012

[hbm70099-bib-0025] Nemati, S. S. , L. Sadeghi , G. Dehghan , and N. Sheibani . 2023. “Lateralization of the Hippocampus: A Review of Molecular, Functional, and Physiological Properties in Health and Disease.” Behavioural Brain Research 454: 114657. 37683813 10.1016/j.bbr.2023.114657

[hbm70099-bib-0026] Nicolaisen‐Sobesky, E. , A. Mihalik , S. Kharabian‐Masouleh , et al. 2022. “A Cross‐Cohort Replicable and Heritable Latent Dimension Linking Behaviour to Multi‐Featured Brain Structure.” Communications Biology 5, no. 1: 1297. 36435870 10.1038/s42003-022-04244-5 PMC9701210

[hbm70099-bib-0027] Nordin, K. , J. Persson , E. Stening , A. Herlitz , E. M. Larsson , and H. Soderlund . 2018. “Structural Whole‐Brain Covariance of the Anterior and Posterior Hippocampus: Associations With Age and Memory.” Hippocampus 28, no. 2: 151–163. 29171897 10.1002/hipo.22817

[hbm70099-bib-0028] Oyarce, D. A. E. , M. E. Shaw , K. Alateeq , and N. Cherbuin . 2020. “Volumetric Brain Differences in Clinical Depression in Association With Anxiety: A Systematic Review With Meta‐Analysis.” Journal of Psychiatry and Neuroscience 45, no. 6: 406–429. 32726102 10.1503/jpn.190156 PMC7595741

[hbm70099-bib-0029] Plachti, A. , S. B. Eickhoff , F. Hoffstaedter , et al. 2019. “Multimodal Parcellations and Extensive Behavioral Profiling Tackling the Hippocampus Gradient.” Cerebral Cortex 29, no. 11: 4595–4612. 30721944 10.1093/cercor/bhy336 PMC6917521

[hbm70099-bib-0030] Plachti, A. , S. Kharabian , S. B. Eickhoff , et al. 2020. “Hippocampus Co‐Atrophy Pattern in Dementia Deviates From Covariance Patterns Across the Lifespan.” Brain 143, no. 9: 2788–2802. 32851402 10.1093/brain/awaa222 PMC7523701

[hbm70099-bib-0031] Plachti, A. , R. D. Latzman , S. M. Balajoo , et al. 2023. “Hippocampal Anterior‐ Posterior Shift in Childhood and Adolescence.” Progress in Neurobiology 225: 102447. 36967075 10.1016/j.pneurobio.2023.102447 PMC10185869

[hbm70099-bib-0032] Poloni, K. M. , I. A. D. de Oliveira , R. Tam , R. J. Ferrari , and Initiative AsDN . 2021. “Brain MR Image Classification for Alzheimer's Disease Diagnosis Using Structural Hippocampal Asymmetrical Attributes From Directional 3‐D Log‐Gabor Filter Responses.” Neurocomputing 419: 126–135.

[hbm70099-bib-0033] Popova, S. , M. E. Charness , L. Burd , et al. 2023. “Fetal Alcohol Spectrum Disorders.” Nature Reviews Disease Primers 9, no. 1: 11. 10.1038/s41572-023-00420-x 36823161

[hbm70099-bib-0034] Reid, A. T. , D. Bzdok , S. Genon , et al. 2016. “ANIMA: A Data‐Sharing Initiative for Neuroimaging Meta‐Analyses.” NeuroImage 124: 1245–1253. 26231246 10.1016/j.neuroimage.2015.07.060

[hbm70099-bib-0035] Schaefer, A. , R. Kong , E. M. Gordon , et al. 2018. “Local‐Global Parcellation of the Human Cerebral Cortex From Intrinsic Functional Connectivity MRI.” Cerebral Cortex 28, no. 9: 3095–3114. 28981612 10.1093/cercor/bhx179 PMC6095216

[hbm70099-bib-0036] Tian, Y. , D. S. Margulies , M. Breakspear , and A. Zalesky . 2020. “Topographic Organization of the Human Subcortex Unveiled With Functional Connectivity Gradients.” Nature Neuroscience 23, no. 11: 1421–1432. 32989295 10.1038/s41593-020-00711-6

[hbm70099-bib-0037] Van Essen, D. C. , S. M. Smith , D. M. Barch , et al. 2013. “The WU‐Minn Human Connectome Project: An Overview.” NeuroImage 80: 62–79. 23684880 10.1016/j.neuroimage.2013.05.041 PMC3724347

[hbm70099-bib-0038] Woolard, A. A. , and S. Heckers . 2012. “Anatomical and Functional Correlates of Human Hippocampal Volume Asymmetry.” Psychiatry Research: Neuroimaging 201, no. 1: 48–53. 10.1016/j.pscychresns.2011.07.016 PMC3289761 22285719

