# Supplementary material for: Discovery, Replicability, and Generalizability of a Left Anterior Hippocampus' Morphological Network Linked to Self‐Regulation
Source: Hum Brain Mapp. 2024 Dec 20;45(18):e70099. doi: 10.1002/hbm.70099 (PMC11661003; doi:10.1002/hbm.70099)
Supplement: Supplementary file 1 — Data S1. [file HBM-45-e70099-s001.pdf]

Supplementary Materials

Discovery, replicability, and generalizability of a left anterior hippocampus' morphological network linked to self-regulation

Somayeh Maleki Balajoo[1, 2], Anna Plachti[1, 2], Eliana Nicolaisen-Sobesky[1, 2], Debo Dong[2, 3], Felix Hoffstaedter[1, 2], Sven G. Meuth[4], Nico Melzer[4], Simon B. Eickhoff[1, 2], Sarah Genon[1, 2]

[1]Institute of Systems Neuroscience, Heinrich Heine University Düsseldorf, Düsseldorf, Germany

[2]Institute of Neuroscience and Medicine (INM-7: Brain and Behaviour), Research Centre Jülich, Jülich, Germany

[3]Key Laboratory of Cognition and Personality, Ministry of Education, Faculty of Psychology, Southwest University, Chongqing, China

[4]Department of Neurology, Medical Faculty and University Hospital Düsseldorf, Heinrich Heine University Düsseldorf, Düsseldorf, Germany

Supplementary Methods

Supplementary results

Cross-Cohort Replicability and Generalizability of the First Latent Dimension

The first latent dimension highlighted a consistent pattern of variability across hippocampal-brain-behavior relationships, demonstrating replicability and generalizability across different cohorts (see Figure S1). To assess the robustness of these findings, we evaluated the consistency of effect sizes, represented by canonical correlation magnitudes, between the discovery and validation cohorts. In both the primary and bidirectional analyses, significant effect sizes were observed in the discovery and validation cohorts, indicating stable relationships (Figure S1).

In the primary analysis, with HCP-YA as the discovery cohort, the effect size of the significant canonical correlation in the second latent dimension was 0.95, while in the validation cohort (HCP-A), this effect size was 0.85 when projected onto the same latent dimension (Figure S1-A). In the bidirectional analysis, where HCP-A served as the discovery cohort and HCP-YA as the validation cohort, the significant canonical correlation effect sizes in the second latent dimension were 0.88 and 0.84, respectively (Figure S1-B). This latent dimension encapsulates a general covariance structure among the hippocampus, the broader brain, and behavioral measures (Figures S2 and S3).

Additionally, in the primary analysis, strong positive correlations were observed between cohorts for both Multi-block and brain loadings, with correlations of $r = 0.95$ ($p_{corr} < .001$) and $r = 0.94$ ($p_{corr} < .001$), respectively. Figure S2 presents brain loading correlations adjusted for the auto-spatial correlation within brain data. Similarly, in the bidirectional analysis, strong positive correlations persisted between cohorts, with $r = 0.98$ ($p_{corr} < .001$) and $r = 0.89$ ($p_{corr} < .001$) for Multi-block and brain loadings, respectively (Figure S3). This

consistency in findings supports the cross-cohort generalizability and robustness of the hippocampal-brain-behavior variability observed.

Cross-Cohort Replicability and Generalizability of the Third and Fourth Latent Dimensions in Primary Analysis

In the primary analysis, using HCP-YA as the discovery cohort and HCP-A as the validation cohort, we identified four significant latent dimensions. Our focus was exclusively on those latent dimensions where all outer splits exhibited significant canonical correlations. While the first two dimensions have already been discussed, we now summarize the third and fourth latent dimensions.

Third Latent Dimension:

For the third latent dimension, the effect size of the identified significant canonical correlation was 0.73 in the discovery cohort, while in the validation cohort (HCP-A), it projected to an effect size of 0.46 (Figure S4-A). This dimension (Figure S4) revealed a positive covariance pattern associated with the head of the right hippocampus, specifically with gray matter volume in the right amygdala. In contrast, it exhibited a negative covariance pattern with the bilateral thalamus, caudate, and overall bilateral cerebral cortex, with the most pronounced effects observed in the dorsolateral prefrontal cortex, inferior and superior parietal lobules, and precuneus. At the behavioral level, this regional-hippocampal-brain covariance pattern demonstrated positive associations with memory, information processing, and higher-order cognitive controls such as emotion regulation and executive function. Conversely, it was negatively correlated with aggressive behavior, stress, hostility/cynicism, anxiety, sleep problems, self-regulation, and life satisfaction. Strong positive correlations between the multi-block and brain loadings across both cohorts were observed, with $r = 0.78$, $p_{corr} < .001$;

$r = 0.68$, $p_{corr} < .001$, respectively (see Figure S4-B and C for brain loading correlations adjusted for the auto-spatial correlation of the brain data).

Fourth Latent Dimension:

In the fourth latent dimension, the effect size of the identified significant canonical correlation was 0.64 in the discovery cohort, while it projected to 0.44 in the validation cohort (HCP-A) (Figure S5-A). This dimension (Figure S5) highlighted the heterogeneity in hippocampal-brain-behavior relationships, differentiating between bilateral CA subregions and bilateral subiculum subregions. Within this morphological network, the bilateral CA subregions displayed a positive covariance pattern with gray matter volume in the bilateral thalamus, globus pallidus, putamen, and various cortical regions associated with parietal and temporal-associative, somatosensory and motor, and visual networks. Conversely, the bilateral CA subregions showed a negative covariance pattern with gray matter volume in the left amygdala, caudate, and cortical regions in the inferior and medial parietal areas, medial prefrontal cortex, and precuneus. At the behavioral level, this regional-hippocampal CA-brain covariance pattern demonstrated positive associations with aggressive behavior, stress, hostility/cynicism, anxiety, sleep problems, fear, and sadness. In contrast, it was negatively correlated with emotion recognition, self-efficacy, purpose, friendship, and life satisfaction. Similarities between the multi-block and brain loadings across the two cohorts in the fourth latent dimension were also evident, with $r = 0.72$, $p_{corr} < .001$; $r = 0.69$, $p_{corr} < .001$,respectively (see Figure S5-B and C for brain loading correlations adjusted for the auto-spatial correlation of the brain data).

Scanning sites effects in the latent dimensions in HCP-A cohort

The analyses linking the morphological network of hippocampal subregions with behavior in the HCP-A cohort, while adjusting for scanning site effects as well as other confounds (age, age-squared, gender, and TIV), identified two significant latent dimensions consistently across

all outer splits, demonstrating robust correlations and statistical significance (Figure S6). Both identified latent dimensions were highly correlated with those derived without adjusting for the scanning site (first latent dimension: $r = 0.99$, $p << 0.0001$; second latent dimension: $r = 0.95$, $p << 0.0001$).

Supplementary Figures

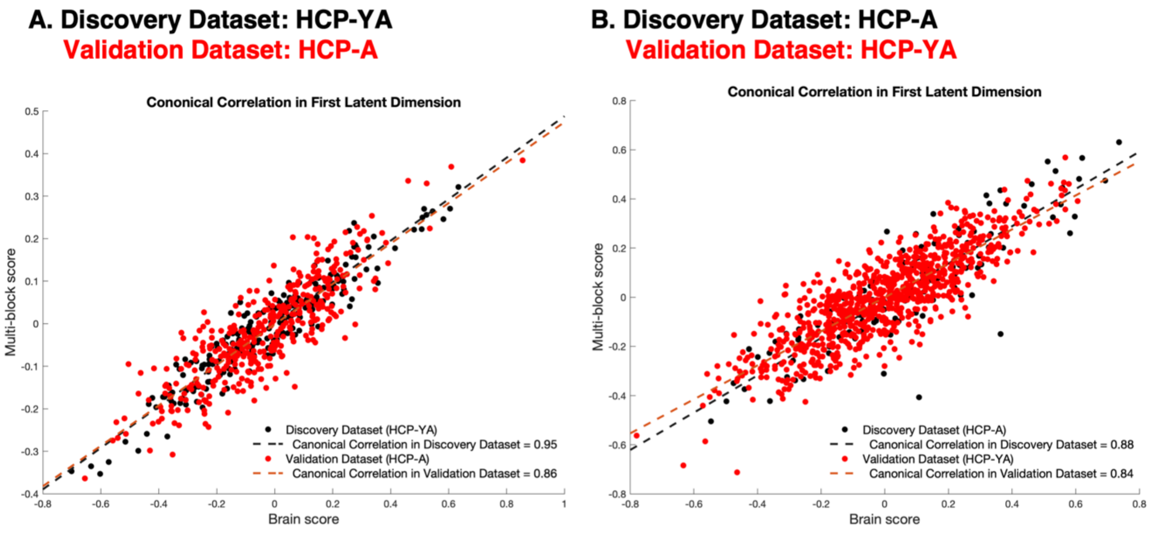

Figure S1. Cross-Cohort Replicability and Generalizability of the First Latent Dimension. This figure illustrates the effect sizes of canonical correlations for the first latent dimension in both discovery and validation cohorts. (A) In the primary analysis, using the HCP-YA as the discovery cohort, the effect size for the significant canonical correlation was 0.95, while the effect size in the validation cohort (HCP-A) was 0.86. (B) In the replication analysis, where HCP-A served as the discovery cohort and HCP-YA as the validation cohort, the effect sizes were 0.88 and 0.84, respectively. All effect sizes are significant, indicating robust relationships between hippocampal-brain-behavior variability across cohorts.

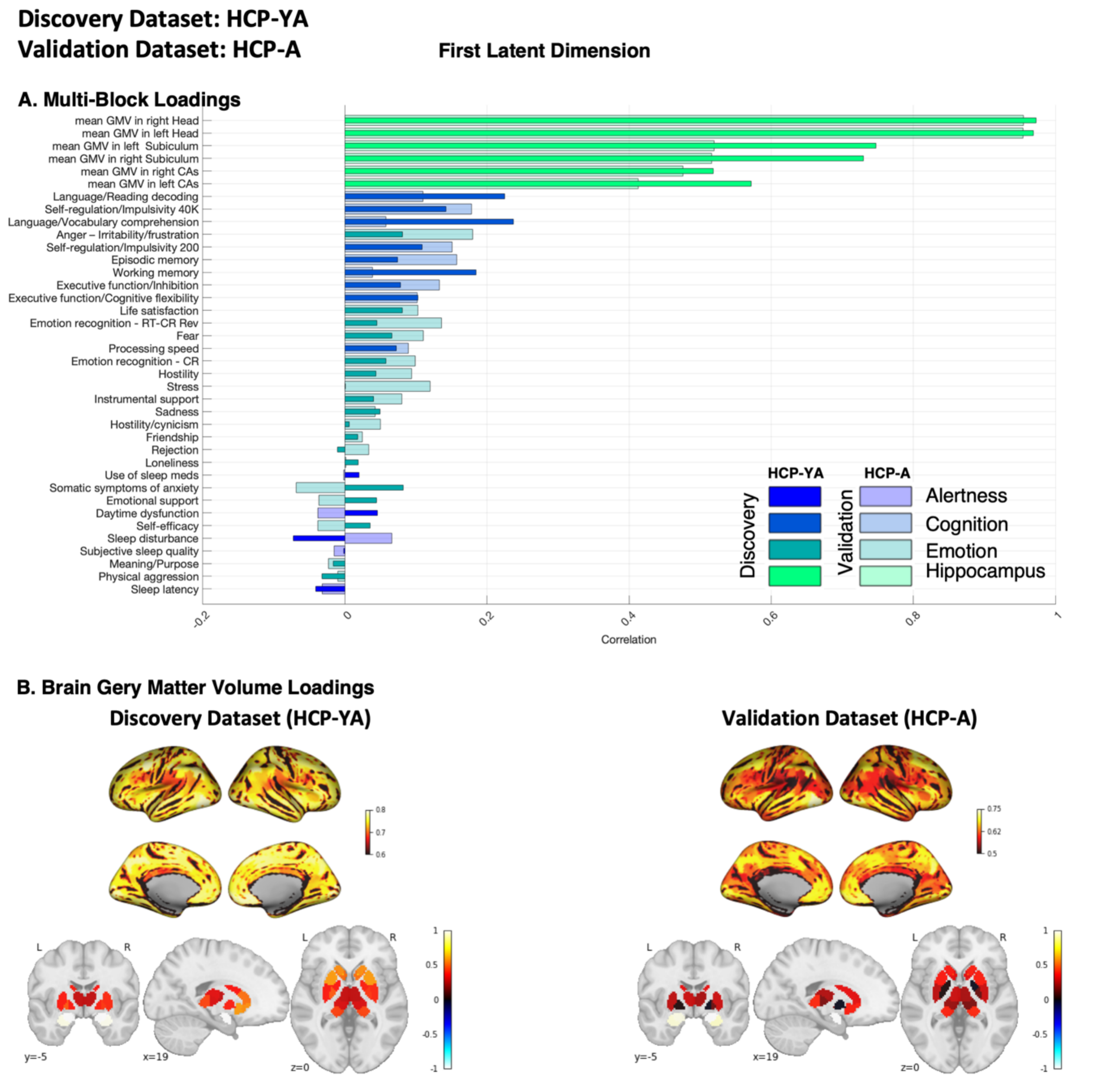

Figure S2. First Latent Dimension in the HCP Young Adults' Cohort. A. Multi-block Loadings; and B. Brain Grey Matter Volume Loadings; A. The shown loadings are averaged across five outer splits, with error bars representing standard deviations. The color-map bars illustrate multi-block variables associated with various domains such as alertness, cognition, emotion, and hippocampal subregions. B. Cortical and subcortical patterns of brain loadings are shown separately for visualization purpose. The subcortical slice corresponds to MNI coordinates: 19, -5, 0. Loadings shown represent the average across the five outer splits, with red indicating positive loadings and blue indicating negative loadings.

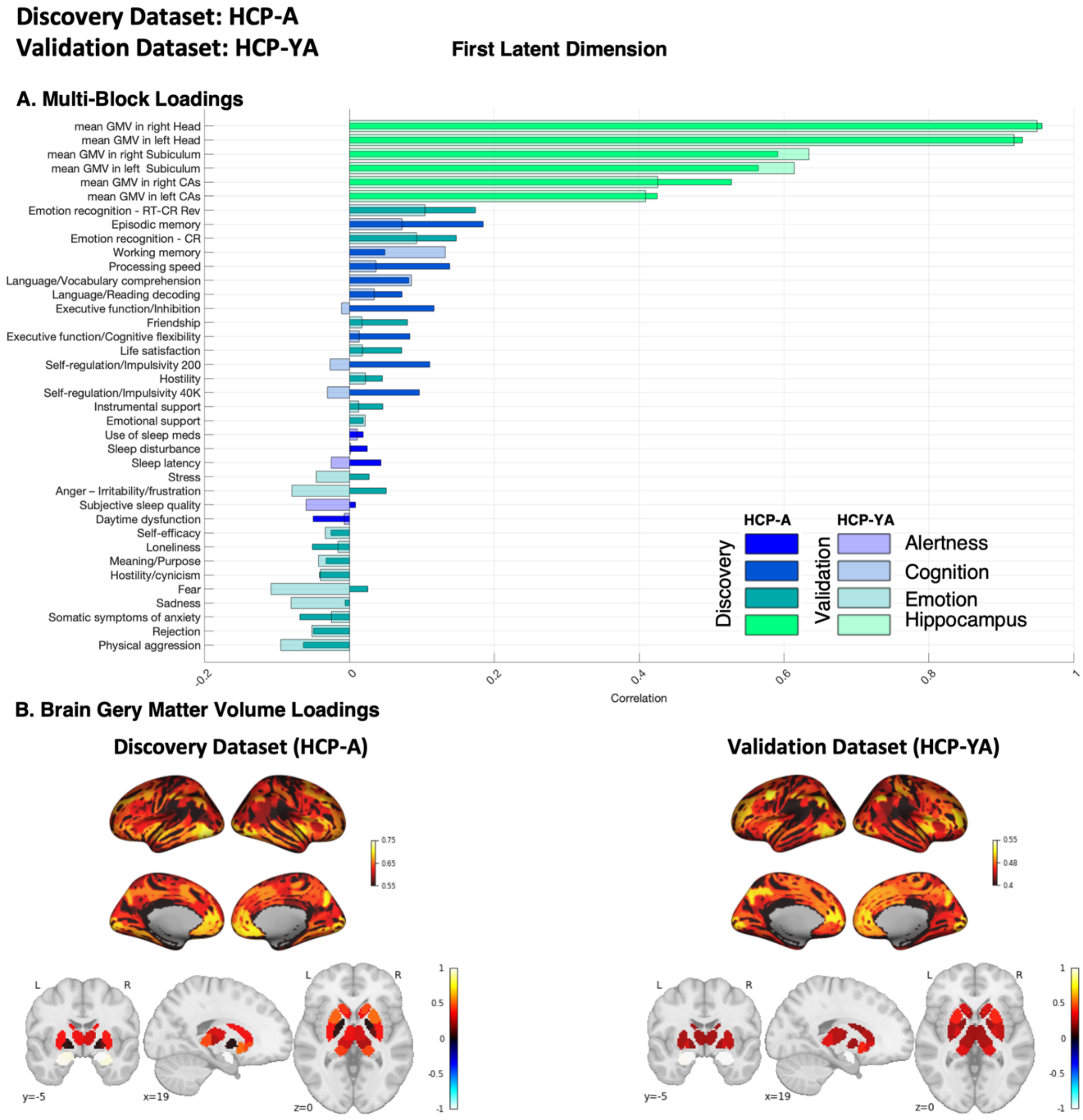

Figure S3. First Latent Dimension in the HCP Aging's Cohort. A. Multi-block Loadings; and B. Brain Grey Matter Volume Loadings; A. The shown loadings are averaged across five outer splits, with error bars representing standard deviations. The color-map bars illustrate multi-block variables associated with various domains such as alertness, cognition, emotion, and hippocampal subregions. B. Cortical and subcortical patterns of brain loadings are shown separately for visualization purpose. The subcortical slice corresponds to MNI coordinates: 19, -5, 0. Loadings shown represent the average across the five outer splits, with red indicating positive loadings and blue indicating negative loadings.

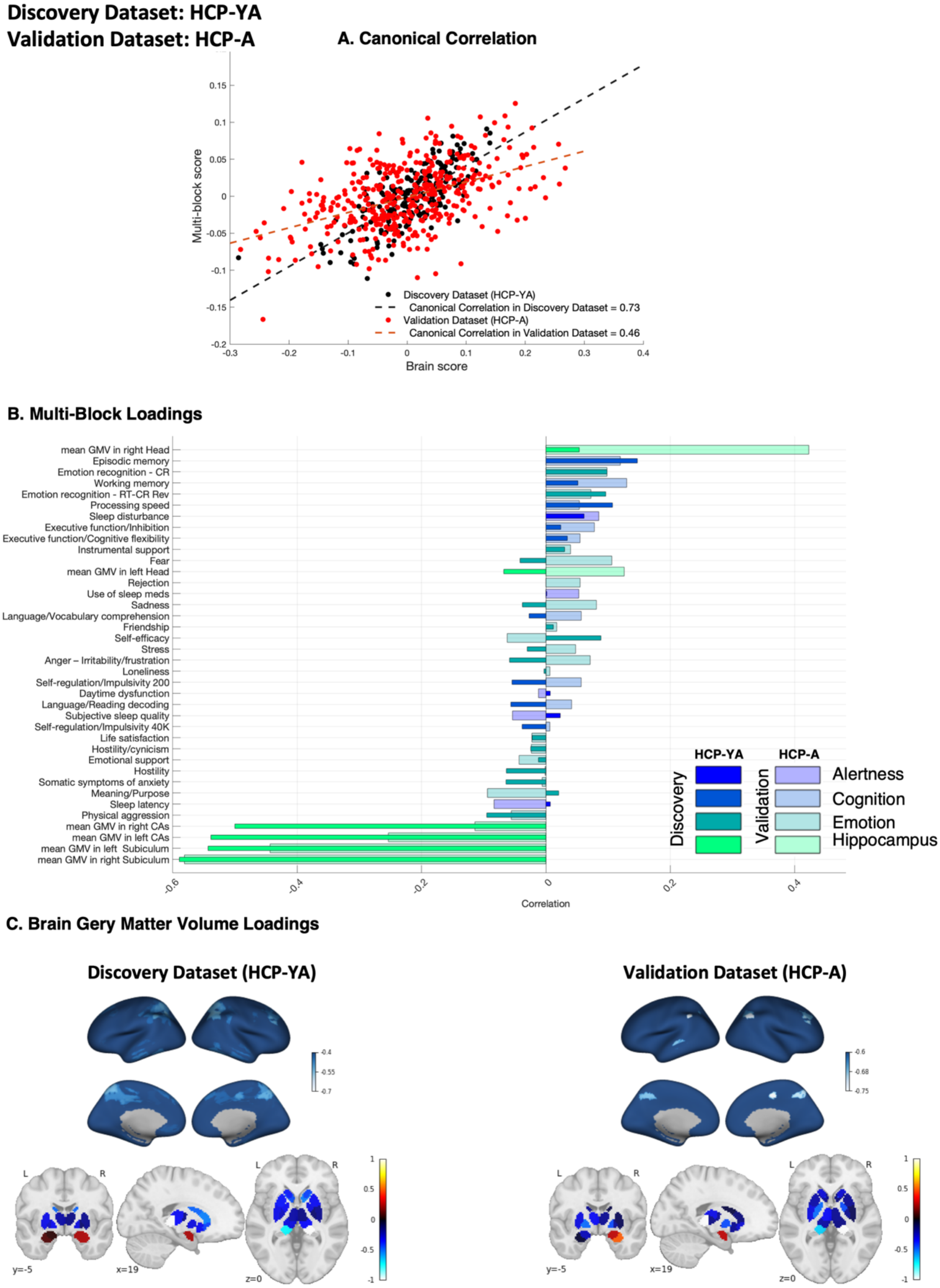

Figure S4. Third Latent Dimension in the HCP Young Adults' Cohort. A. Multi-block Loadings; and B. Brain Grey Matter Volume Loadings; A. The shown loadings are averaged across five outer splits, with error bars representing standard deviations. The color-map bars illustrate multi-block variables associated with various domains such as alertness, cognition, emotion, and hippocampal subregions. B. Cortical and subcortical patterns of brain loadings are shown separately for visualization purpose. The subcortical slice corresponds to MNI coordinates: 19, -5, 0. Loadings shown represent the average across the five outer splits, with red indicating positive loadings and blue indicating negative loadings.

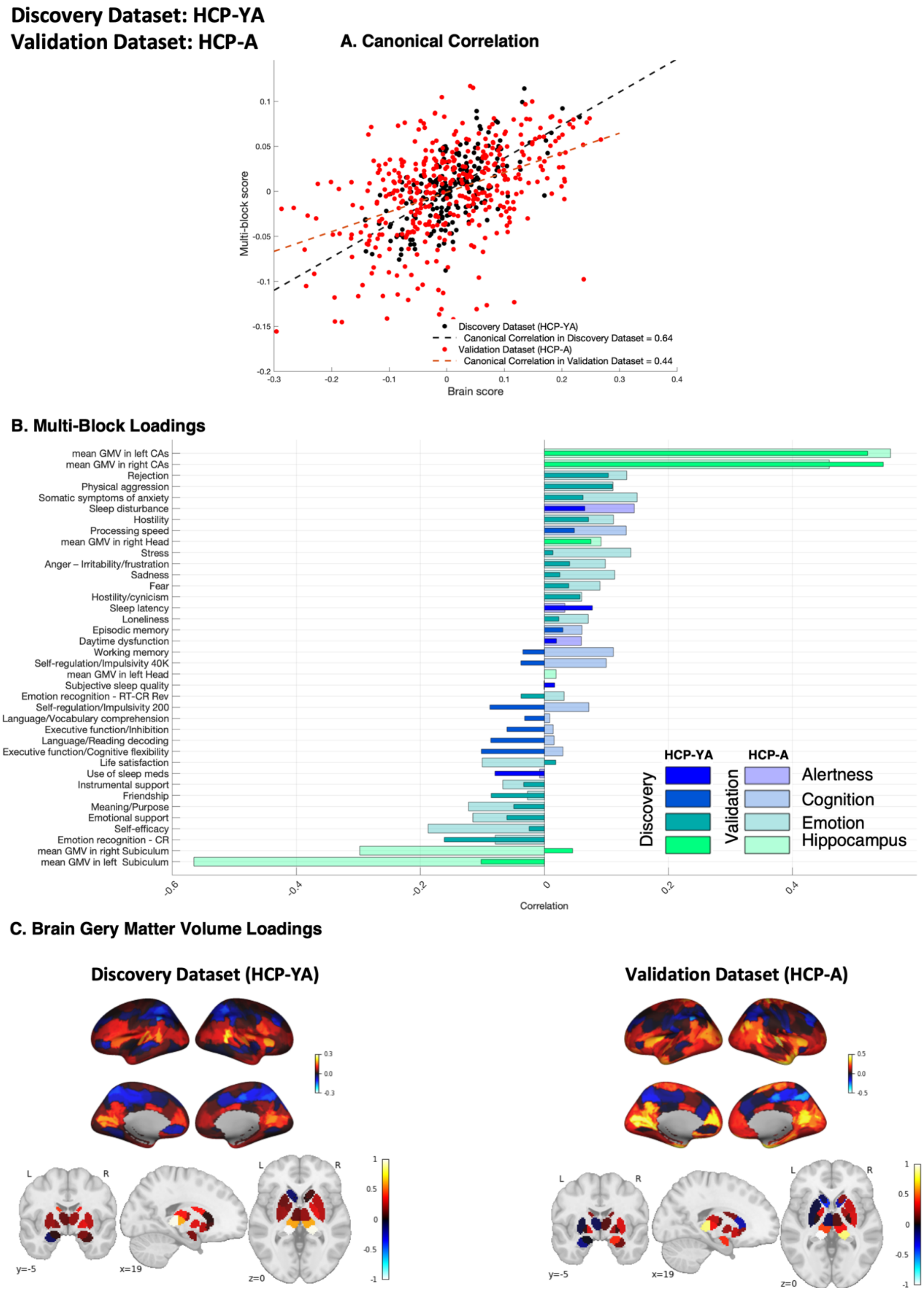

Figure S5. Forth Latent Dimension in the HCP Young Adults' Cohort. A. Multi-block Loadings; and B. Brain Grey Matter Volume Loadings; A. The shown loadings are averaged across five outer splits, with error bars representing standard deviations. The color-map bars illustrate multi-block variables associated with various domains such as alertness, cognition, emotion, and hippocampal subregions. B. Cortical and subcortical patterns of brain loadings are shown separately for visualization purpose. The subcortical slice corresponds to MNI coordinates: 19, -5, 0. Loadings shown represent the average across the five outer splits, with red indicating positive loadings and blue indicating negative loadings.

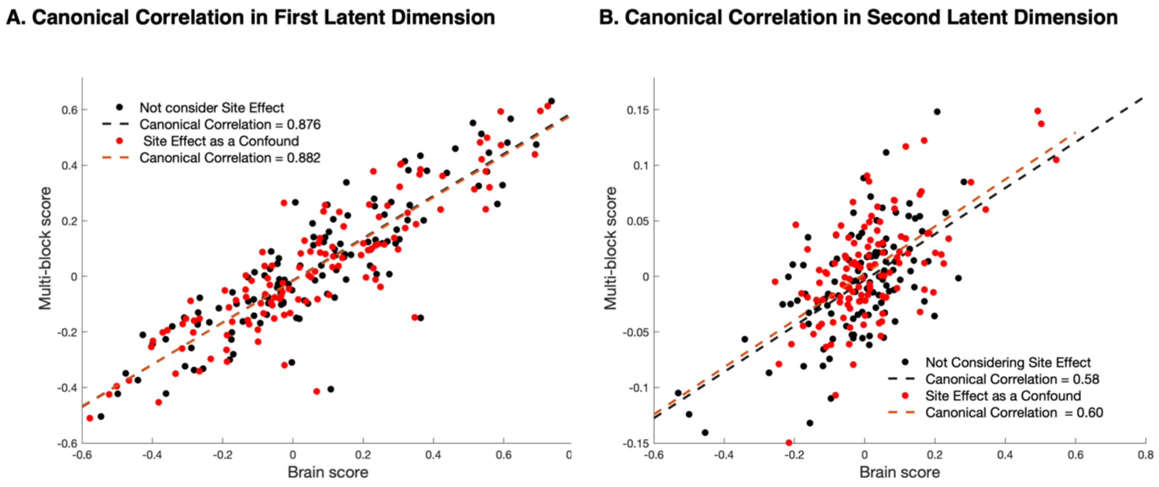

Figure S6. Scanning site effect in the HCP-A cohort. A. Canonical correlation in first latent dimension; B. Canonical correlation in second latent dimension. Latent dimensions linking the morphological network of hippocampal subregions with behavior in the HCP-A cohort appear unaffected by potential confounding effects from the scanning site. Both latent dimensions, adjusted for the scanning site, were highly correlated with those derived without adjusting for scanning site (first latent dimension: $r = 0.99$, $p << 0.0001$; second latent dimension: $r = 0.95$, $p << 0.0001$).

Supplementary Tables

S1 Table. Behavioral variables.

Category/ Domain	Subdomain	Column Header	Measure name	Label
Alertness	Sleep	PSQI_Comp1	PSQI	Subjective sleep quality1
		PSQI_Comp2	PSQI	Sleep latency
		PSQI_Comp5	PSQI	Sleep disturbance
		PSQI_Comp6	PSQI	Use of sleep meds
		PSQI_Comp7	PSQI	Daytime dysfunction
Cognition	Episodic memory	PicSeq_Unadj	Picture sequence memory	Episodic memory
	Executive function/Cognitive flexibility	CardSort_Unadj	Dimensional change card sort	Executive function/Cognitive flexibility
	Executive function/Inhibition	Flanker_Unadj	Flanker inhibitory control and attention task	Executive function/Inhibition
	Language/Reading decoding	ReadEng_Unadj	Oral reading recognition	Language/Reading decoding
	Language/Vocabulary comprehension	PicVocab_Unadj	Picture vocabulary	Language/Vocabulary comprehension
	Processing speed	ProcSpeed_Unadj	Pattern comparison processing speed	Processing speed
	Self-regulation/Impulsivity	DDisc_AUC_200	Delay discounting	Self-regulation/Impulsivity1
		DDisc_AUC_40K	Delay discounting	Self-regulation/Impulsivity2
	Working memory	ListSort_Unadj	List sorting	Working memory
Emotion	Emotion recognition	ER40_CR	Penn emotion recognition test	Emotion recognition - CR
		ER40_CRT	Penn emotion recognition test	Emotion recognition - RT-CR Rev
	Negative Affect	AngAffect_Unadj	NIH Toolbox Anger-Affect Survey	Anger - Irritability/frustration
		AngHostil_Unadj	NIH Toolbox Anger-Hostility Survey	Hostility/cynicism
		AngAggr_Unadj	NIH Toolbox Anger-Physical Aggression Survey	Physical aggression
		FearAffect_Unadj	NIH Toolbox Fear-Affect Survey	Fear
		FearSomat_Unadj	NIH Toolbox Fear-Somatic Arousal Survey	Somatic symptoms of anxiety
		Sadness_Unadj	NIH Toolbox Sadness Survey	Sadness
	Psychological well-being	LifeSatisf_Unadj	NIH Toolbox General Life Satisfaction Survey	Life satisfaction
		MeanPurp_Unadj	NIH Toolbox Meaning and Purpose Survey	Meaning/Purpose
	Social relationships	Friendship_Unadj	NIH Toolbox Friendship Survey	Friendship
		Loneliness_Unadj	NIH Toolbox Loneliness Survey	Loneliness
		PercHostil_Unadj	NIH Toolbox Perceived Hostility Survey	Hostility

		PercReject_Unadj	NIH Toolbox Perceived Rejection Survey	Rejection
		EmotSupp_Unadj	NIH Toolbox Emotional Support Survey	Emotional support
		InstruSupp_Unadj	NIH Toolbox Instrumental Support Survey	Instrumental support
	Stress and Self Efficacy	PercStress_Unadj	NIH Toolbox Perceived Stress Survey	Stress
		SelfEff_Unadj	NIH Toolbox Self-Efficacy Survey	Self-efficacy

ASR: Achenbach Adult Self-Report / PSQI: Pittsburgh Sleep Quality Index´

S2 Table. Latent dimensions in the HCP-YA dataset.

Level	Split	Canonical correlation (r coefficient)	p-value uncorrected	p-value corrected
1	1	0.93	0.001	0.005
	2	0.94	0.001	0.005
	3	0.95	0.001	0.005
	4	0.93	0.001	0.005
	5	0.94	0.001	0.005
2	1	0.69	0.001	0.005
	2	0.70	0.001	0.005
	3	0.71	0.001	0.005
	4	0.66	0.001	0.005
	5	0.72	0.001	0.005
3	1	0.65	0.001	0.005
	2	0.73	0.001	0.005
	3	0.69	0.001	0.005
	4	0.66	0.001	0.005
	5	0.72	0.001	0.005
4	1	0.64	0.001	0.005
	2	0.50	0.001	0.005
	3	0.51	0.001	0.005
	4	0.52	0.001	0.005
	5	0.48	0.001	0.005

Statistical results for the significant latent dimension are shown for each one of the 5 outer splits. P-values are shown as uncorrected and corrected for multiple comparisons using the Bonferroni method over 5 comparisons (corresponding to the 5 outer splits) [2]. Asterisks indicate splits that yielded significant latent dimensions. r: Pearson's correlation. In each level, best split highlighted with gray.

S3 Table. Latent dimensions in the HCP-A dataset.

Level	Split	Canonical correlation (r coefficient)	p-value uncorrected	p-value corrected
1	1	0.88	0.001	0.005
	2	0.85	0.001	0.005
	3	0.85	0.001	0.005
	4	0.85	0.001	0.005
	5	0.85	0.001	0.005
2	1	0.41	0.001	0.005
	2	0.49	0.001	0.005
	3	0.57	0.001	0.005
	4	0.59	0.001	0.005
	5	0.52	0.001	0.005

Statistical results for the two significant latent dimensions are shown for each one of the 5 outer splits. P-values are shown as uncorrected and corrected for multiple comparisons using the Bonferroni method over 5 comparisons (corresponding to the 5 outer splits) [2]. Asterisks indicate splits that yielded significant latent dimensions. r: Pearson's correlation. In each level, best split highlighted with gray.
